# Contrasting morphology with molecular data: an approach to revision of species complexes based on the example of European *Phoxinus* (Cyprinidae)

**DOI:** 10.1186/s12862-017-1032-x

**Published:** 2017-08-09

**Authors:** Anja Palandačić, Alexander Naseka, David Ramler, Harald Ahnelt

**Affiliations:** 1 First Zoological Department, Vienna Museum of Natural History, Burgring 7, 1010 Vienna, Austria; 2 Department of Limnology and Bio-Oceanography, University of Vienna, Althanstrasse 14, 1090 Vienna, Austria; 3 Department of Theoretical Biology, University of Vienna, Althanstrasse 14, 1090 Vienna, Austria; 4 Department of Ichthyology and Hydrobiology, Faculty for Biology and Soil, Saint Petersburg State University, 7/9 Universitetskaya nab, St. Petersburg, 199034 Russia

**Keywords:** Cryptic diversity, Species delimitation, Molecular taxonomy, *Phoxinus* (Cyprinidae)

## Abstract

**Background:**

Molecular taxonomy studies and barcoding projects can provide rapid means of detecting cryptic diversity. Nevertheless, the use of molecular data for species delimitation should be undertaken with caution. Especially the single-gene approaches are linked with certain pitfalls for taxonomical inference. In the present study, recent and historical species descriptions based upon morphology were used as primary species hypotheses, which were then evaluated with molecular data (including in type and historical museum material) to form secondary species hypotheses. As an example of cryptic diversity and taxonomic controversy, the European *Phoxinus phoxinus* species complex was used.

**Results:**

The results of the revision showed that of the fourteen primary species hypotheses, three were rejected, namely *P. ketmaieri*, *P. likai*, and *P. apollonicus*. For three species (*P. strandjae*, *P. strymonicus, P. morella*), further investigation with increased data sampling was suggested, while two primary hypotheses, *P. bigerri* and *P. colchicus*, were supported as secondary species hypotheses. Finally, six of the primary species hypotheses (*P. phoxinus*, *P. lumaireul*, *P. karsticus*, *P. septimanae*, *P. marsilii* and *P. csikii*) were well supported by mitochondrial but only limitedly corroborated by nuclear data analysis.

**Conclusion:**

The approach has proven useful for revision of species complexes, and the study can serve as an overview of the *Phoxinus* genus in Europe, as well as a solid basis for further work.

**Electronic supplementary material:**

The online version of this article (doi:10.1186/s12862-017-1032-x) contains supplementary material, which is available to authorized users.

## Background

The current expansion in the destruction of ecosystems and species extinction calls for prompt biodiversity assessment. However, biodiversity estimates are influenced strongly by the existence of cryptic species, which are—according to Bickford et al. [1] in a definition adopted in the present study—two or more species classified as a single nominal species as they are (cursorily) morphologically indistinguishable. It has become clear from molecular data that cryptic species are common and found throughout all metazoan taxa [2, 3]. Although cryptic diversity is not necessarily a consequence of a lack of morphological differences between taxa, and can result from a deficiency of appropriate taxonomic studies, molecular taxonomy studies and barcoding projects have provided a quick and efficient means for uncovering cryptic diversity [4, 5].

However, using such methods for species delimitation and final taxonomic implication is not without problems and should be utilised with caution [6]. Especially the barcoding method, which is a single-gene approach, is linked with certain pitfalls for taxonomical inference such as introgression and/or incomplete lineage sorting [7, 8]. Thus, additional sampling of one or more unlinked genes, morphological characters, ecological factors and/or geographic distributions are to be used to complement the phylogeny of the barcoding gene in the species delimitation process [9].

In fishes, a literature survey performed by Pérez-Ponce de Leon and Poulin [10], reported the existence of 468 cryptic species that can be found in well studied genera [11]. In the European *Phoxinus* species*,* morphological characters generally used by traditional taxonomy (Additional file 1: Table S1) seem to offer limited phylogenetic information for resolving interspecies relationships and morphological studies disagree about the validity of some of the putative species within the genus (e.g., validity of *P. lumaireul*; [12, 13] vs. [14]). Additionally, morphometric geometric studies of *Phoxinus* have demonstrated a plasticity in body shape dependent upon habitat [15, 16], which influences some of the characters proposed for species delimitation (e.g., eye diameter, caudal peduncle depth). Thus, lack of obvious morphological characters and further diversity in the genus *Phoxinus* revealed by molecular studies [17, 18] point to the existence of cryptic lineages in the genus. At present, eleven *Phoxinus* species are suggested for European drainages (Table 1), with *P. phoxinus* having a very broad distribution that includes the north-eastern Atlantic, North Sea, Baltic, and Black Sea basins [12, 19]. Kottelat [13] and Kottelat & Freyhof [12] mentioned the Danube minnow as a lineage different from *P. phoxinus*, though they gave no morphological characteristics enabling such discrimination. Knebelsberger et al. [17] and Palandačić et al. [18] confirmed the existence of several genetic lineages in the Danube drainage, but did not connect any of the lineages with the available names: *P. csikii* Hankó, 1922 from northern Montenegro and *P. marsilii* Heckel, 1836 from the vicinity of Vienna. Kottelat [13] included both names in the synonymy of *P. phoxinus*.

**Table 1:** Primary species vs. secondary species hypotheses

Primary species hypothesis			Secondary species hypothesis				
From the literature			Result of this study				
Species (*Phoxinus*)	Species range	Remarks	Clade	mtDNA	ntDNA	Adjusted range	Remarks
*P. apollonicus* Bianco & De Bonis, 2015 [19]	River Morača and its tributary Zeta, belonging to the Skadar Lake basin		7	synonym of *P. karsticus*			
*P. bigerri* Kottelat, 2007 [12]	Adour drainage, Ebro drainage, streams of Cantabric range, introduced in Douro drainage		13	confirmed	confirmed	Ugarna drainage, Bay of Biscay	Indication of two species, other species with distribution ranges Adour and Ebro drainages
*P. colchicus* Berg, 1910	According to Kottelat [9]: rivers in Ozurgety District, Black Sea	Elevated to species level Bogutskaya & Naseka [62]; Kottelat & Freyhof [12] possibly two species	18	confirmed	confirmed	Natanebi drainage, Black Sea	Indication of two species confirmed; other species Mchishta drainage, Black Sea
*P. karsticus* Bianco & De Bonis, 2015 [19]	Probably endemic to the karstic Popovo Polje–Trebinje endorheic river system		7	confirmed	confirmed	Skadar Lake drainage and some sinking streams, Adriatic Sea	
*P. ketmaieri* Bianco & De Bonis, 2015 [19]	Krk Island, Zrmanja River, probably other rivers of the Dalmatian district (Krka, Neretva)		1	synonym of *P. lumaireul*			Krka and Neretva belong to other clades (see below)
*P. likai* Bianco & De Bonis, 2015 [19]	Probably endemic to the endorheic river system of Lika region		1	synonym of *P. lumaireul*			
*P. lumaireul* (Schinz, 1840)	Po River, Italy, according to Kottelat [9] Adriatic basin from Po to Drin drainages	Originally *Cyprinus lumaireul*, revalidated by Kottelat [13]	1	confirmed	confirmed sensu stricto	North Adriatic Sea basin and middle Danube drainage, Black Sea	
*P. phoxinus* (Linnaeus, 1758)	According to Kottelat [9], basins of Atlantic, North and Baltic Seas	Originally *Cyprinus phoxinus*	10	confirmed	limited support	Rhine drainage, North Sea	According to molecular data very restricted range
*P. strandjae* Drensky, 1926	According to Kottelat [13], Veleka and Resowska drainages, draining from Strandzha range to Black Sea		14	uncertain	limited support	Unchanged	Denser sampling and further analysis needed
*P. strymonicus* Kottelat, 2007 [12, 13]	Struma, possibly Loudias and Filiouris drainages		15	uncertain	no data available	Unchanged	Denser sampling and further analysis needed
*P. septimanae* Kottelat, 2007 [12, 13]	Mediterranean coastal streams from Gardon to Tech		12	confirmed	limited support	Unchanged	
*P. csikii* Hanko, 1922 [61]	Only the type locality given in the description—Korita, Bijelo Polje	According to Kottelat [13], synonym of *P. phoxinus*	5	confirmed	confirmed sensu stricto	Mostly right tributaries of Danube drainage, Black Sea	
*P. marsilii* Heckel, 1836 [60]	Small streams in the surroundings of Vienna and beyond	According to Kottelat [13], synonym of *P. phoxinus*	9	confirmed	limited support	Middle and lower Danube drainage, mostly the left tributaries, Black Sea	
*P. morella* (Leske, 1774)	Only the type locality given in the description: Bode Creek near Rübeland, Germany, Elbe drainage, North Sea	Originally *Cyprinus morella*; according to Kottelat [13], synonym of *P. phoxinus*	11	uncertain	no data available	Elbe and Weser drainages, North Sea, but also Danube	Specimens from the type locality needed
*Phoxinus* sp. 1			2	uncertain	confirmed	Neretva drainage and sinking streams mouthing to middle Adriatic Sea; but also Danube drainage, Black Sea basin	Denser sampling and further analysis needed
*Phoxinus* sp. 2			3	uncertain	limited support	Danube drainage, Black Sea	Denser sampling and further analysis needed
*Phoxinus* sp. 3			4	uncertain	limited support	Danube drainage, Black Sea	Denser sampling and further analysis needed
*Phoxinus* sp. 4			6	confirmed	confirmed	Krka River, Adriatic Sea basin	Well separated from other clades
*Phoxinus* sp. 5			8	confirmed	limited support	Ohrid Lake basin, Adriatic Sea	Well separated from other clades
*Phoxinus* sp. 6			16	uncertain	no data available	Rhone drainage, Mediterranean Sea	Only one specimen with this haplotype found
*Phoxinus* sp. 7			17	confirmed	confirmed	Baltic, North Sea basins (excluding southern coast of the North Sea)	Indication of two species confirmed with ntDNA.

The present study had two aims. The first was to test an approach for revising species complexes, in which morphologically defined species (the validity of which is put into question by contrary research) are considered as primary species hypotheses, which are then evaluated with molecular data to form secondary species hypotheses. Because molecular data for species delimitation should include at least two unlinked genes, but there are often discrepancies between them (e.g., mitochondrial DNA (mtDNA) vs. nuclear DNA (nuDNA)), reasons, consequences and possible solutions for those discrepancies were discussed. The second aim was to revise the European *Phoxinus phoxinus* species complex, as it is an example of cryptic diversity and taxonomic controversy. Recent and historical morphological species descriptions served as a basis for evaluation with available molecular data of *Phoxinus* from previous studies, the International Barcode of Life (iBOL) project, new samples from the Danube drainage, type material of *P. marsilii* and historical material of *Phoxinus* sp. from a locality close to the type locality of *P. csikii*. The revision included linking of new lineages with the available species names, and where necessary taxonomical implications.

## Methods

### Samples and dataset

In previous phylogenetic studies and barcoding projects [17, 18, 20, 21], mostly two mitochondrial genes—the barcoding region of cytochrome oxidase I gene (COI) and cytochrome *b* (cytb)—have been used. Unfortunately, some putative *Phoxinus* species in Europe are only represented by COI. Most importantly, however, the COI region is available for *P. phoxinus* sensu stricto, thus enabling the COI dataset to be used for phylogenetic reconstruction and species delimitation. Among the Ichthyology Collection in the Swedish Museum of Natural History, Stockholm, genetic data of the neotype of *P. phoxinus* (NRM-55108) are not available (the neotype was fixed in formalin). Therefore, COI sequences determined by Knebelsberger et al. [17] as the genetic lineage corresponding to *P. phoxinus* sensu stricto were used for reference in this study. The COI region is very short, thus cytb data (and the combination of the two) were used where available (see also Results section). As cases of hybridization have often been reported in cyprinids [22], molecular analysis included two nuclear genes: rhodopsin and recombination activating gene 1 (RAG1). Both genes were previously used in *Phoxinus* phylogenetic studies; however, rhodopsin was shown to have limited power for species delimitation in this genus [23]. Nevertheless, sequences of otherwise unavailable material were available in the Genbank, therefore rhodopsin was included in the dataset. Similarly, RAG1 also had only limited delimitation capacity [18], thus a much longer segment (1413 bp instead of 841 bp) was used in this study.

**Fig. 1:**
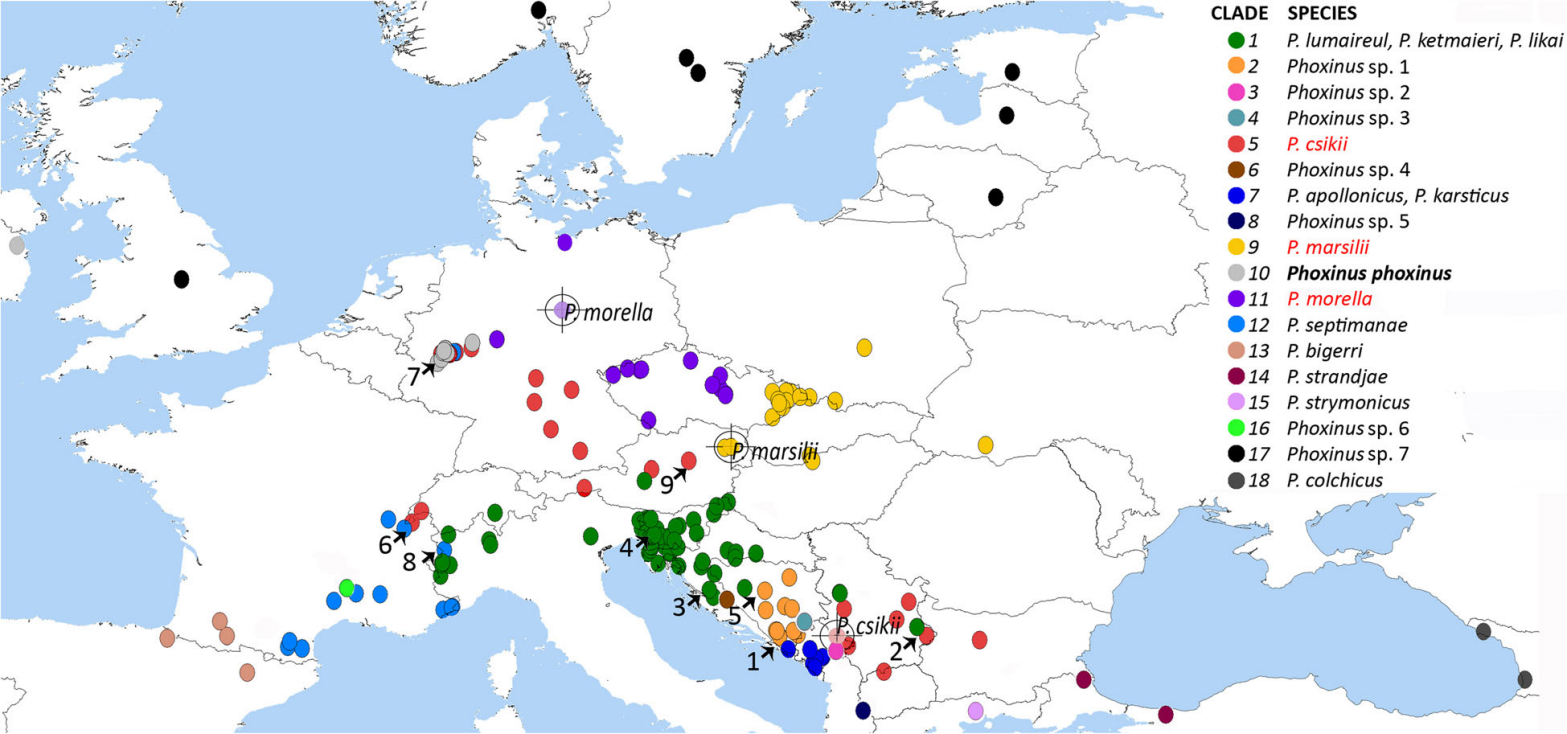
Sampling sites used in this study**.** Data set comprise a combination of new material and data downloaded from Genbank. For details see Additional file 2: Tables S2. Target symbols denote type localities of *Phoxinus csikii*, *P. marsilii* and *P. morella*. We do not have genetic data from the type localities of *P. csikii* and *P. morella* (points marked in *pale red* and *pale violet*, respectively). The *arrows* on the figure mark areas, where hybrids were detected. They correspond to *arrows* in Fig. 3b

All sampling sites, with the exception of KU729260 from Kama River (Russia), are depicted in Fig. 1. The dataset includes all major European drainages combining available molecular data of *Phoxinus* from previous studies and barcoding projects and new samples from the Black, Baltic and Mediterranean Sea basins, collected in this study. All sampling sites are reported in Additional file 2: Table S2 including detailed information such as water body, GPS coordinates and Genbank number where applicable. Procedures for DNA extraction and polymerase chain reaction (PCR) conditions for fresh material are also available in the supplementary material. Sequences were edited by eye and aligned using MEGA 5.0 [24]. Generally, sequences from Genbank were of poor quality, exhibiting numerous ambiguous positions. Where multiple sequences from the same locality were available (e.g., Wahlscheid, Germany [17]), only those without missing data were used for further analysis.

### Museum material

To clarify the taxonomic status of *P. marsilii* Heckel, 1836, the six syntypes registered under NMW-51225 (collection of the Natural History Museum Vienna, NMW, Austria) were included in the study. The whereabouts of the two syntypes of *P. csikii* Hanko, 1922 are unknown (personal communication with J. Vörös curator of the Ichthyology Collection at the Hungarian Natural History Museum); thus museum material collected in the same year as those syntypes (labelled *P. laevis* NMW-51266), from a geographically proximate river, the Ibar at Rožaje, Montenegro, were used in the present study. Finally, old museum material from NMW and ZMB collections (Museum of Natural History Berlin, Germany) from around Germany was included.

Laboratory procedures involving museum material were performed in a DNA clean room with sterilised and UV-irradiated utensils. DNA was extracted from air-dried tissue with QIAamp® DNA Mini and Blood Mini Kit (Qiagen) following manufacturer’s protocol. All extractions included extraction controls to ensure there was no contamination of the buffers. Because museum DNA typically is fragmented, additional primers to amplify from 150 to 350 bp-long fragments of COI and cytb were developed and arranged across the regions in a way that adjacent fragments overlapped for at least 30 bp, an additional control for contamination. For COI, the complete region (652 bp) was put together, while for cytb 590 or 473 bp-long parts were obtained, depending on the DNA quality. Touch-down PCR protocol was used for all fragments, together with a larger number (45) of cycles, and included negative and positive reaction controls. Primers, their lengths and PCR conditions are reported in the supplementary material (Additional file 3: Table S3). PCR products were purified with Qiagen PCR purification kit and sequenced in both directions by LGC Genomics (Berlin, Germany) with PCR primers. Finally, the fragments were aligned using MEGA 5.0 [24] and combined into a single sequence (aligned fasta files in supplementary material). Composed sequences were then added to the dataset for phylogenetic and species delimitation analysis.

### Mitochondrial DNA

#### Phylogenetic analysis

To revise putative *Phoxinus* species in Europe, primarily the COI dataset was used for phylogenetic analysis. Besides, phylogenetic reconstruction was performed from three additional data sets: cytb, COI + cytb and COI + partial cytb region corresponding to the shorter length (475 bp) of the cytb fragment amplified from the museum samples. For all alignments, the most appropriate model of nucleotide substitution was selected using hierarchical likelihood ratio tests implemented by jModelTest v.0.1.1 [25]. Phylogenetic trees were constructed from the alignments using Bayesian inference (BI) with BEAST 1.8.0 [26]. First, an appropriate model for phylogenetic reconstruction for each dataset was determined using path sampling and stepping-stone model selection criteria [27, 28]. Three independent runs were performed and combined with LogCombiner (part of the BEAST package) once the first 10% of steps of each run were discharged as a burn-in phase. Phylogenetic trees were also constructed using the Maximum-Likelihood (ML) method (with an appropriate model of nucleotide substitution) implemented in PhyML [29] and, because the PhyML program does not support partitioning, GARLI 2.01 [30, 31] was used for constructing ML trees for the combined datasets. Because the aim of the study was to determine the number of clades (species) in European *Phoxinus* and not the succession of their splits, unrooted phylogenetic trees were constructed. Genetic distances between and within clades detected in the phylogenetic analysis were calculated in MEGA 5.0 [24] (between group mean distances - the number of base substitutions per site from averaging over all sequence pairs between groups) using an appropriate model of nucleotide substitution (for more details see Results in the supplementary material). To check for possible multiple connections among haplogroups that are not evident when using a strictly bifurcating approach, an unrooted minimum-spanning network was constructed with COI and the median-joining algorithm [32] implemented in Network 5.1 (www.fluxus-engineering.com) with default settings. More information on model selection (Additional file 3: Tables S4 and S5) and phylogenetic analysis can be found in the supplementary material.

#### Species delimitation

To evaluate the clades detected by phylogenetic analysis, species delimitation was performed on the COI dataset using three different methods, each of which employs a different approach for delimiting species. Automatic Barcode Gap Discovery (ABGD; [33]) automatically detects a gap in the distribution of pairwise genetic distances, and two tree-based methods: General Mixed Yule Coalescent model (GMYC; [34]) for ultrametric trees and Poisson Tree Processes (PTP; [35]) for phylogenetic trees not calibrated for time. The details are reported in the supplementary material.

#### Isolation by distance (IBD)

To test if the subclades 1a–1f, 5a, 5b, 9a and 9b detected by phylogenetic and network analysis are a consequence of isolation by distance, a Mantel test correlation using an IBD web service (http://ibdws.sdsu.edu/~ibdws/) with default settings was performed, except that the number of randomizations was increased to 10,000 as suggested by the authors [36]. Genetic distances between populations were calculated in MEGA 5.0 [24] as described above, and plotted against geographic distance calculated with DIVA-GIS 7.5.0 [37].

### Nuclear DNA

#### Haplotype networks

For both nuclear genes, the gametic phase of heterozygous individuals was determined using Phase 2.1 [38, 39], implemented in DnaSP 5.10 [40]. Phase is using a coalescent-based Bayesian algorithm, which has been shown to represent a reliable alternative to cloning [41, 42]. The program was run five times with altered seeds for the random number generator, with 1000 iterations, of which 20% were burn in, and a thinning interval of 10. As suggested by the manual, the consistency of the results was checked by inspection of goodness-of-fit measure across the runs. After the gametic phase was resolved, unrooted minimum-spanning networks were constructed with median-joining algorithm [32] implemented in Network 5.1 (www.fluxus-engineering.com) with default settings. For rhodopsin, one haplotype network was constructed (see Results), while for RAG1, two haplotype networks were produced – one with longer fragment using only data from this study and one shorter with combined data from previous studies.

An overview of the material, genes and analysis used is presented in the Table 2.

**Table 2:**
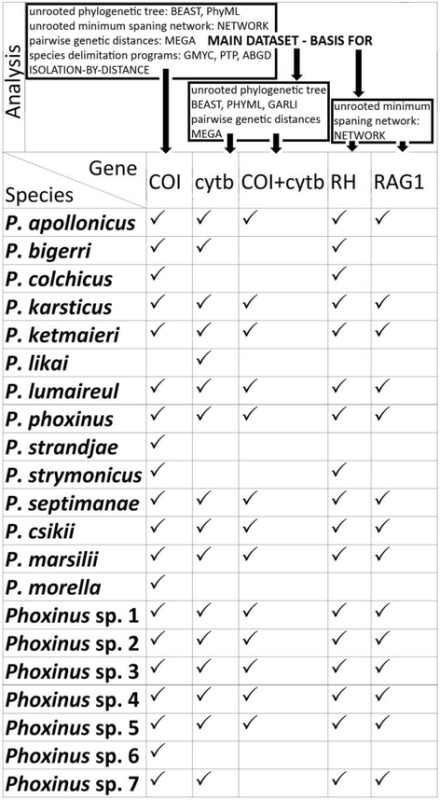
An overview of the material, genes and analysis used is this study

Versions of programs used: BEAST 1.8.0 [26]; PhyML [29], GARLI 2.01 [30, 31], NETWORK 5.0 (www.fluxus-engineering.com), MEGA 5.0 [24]; species delimitation programs: *ABGD* Automatic Barcode Gap Discovery [33], *GMYC* General Mixed Yule Coalescent model [34], *PTP* Poisson Tree Processes [35]; ISOLATION-BY-DISTANCE calculated in IBD web service (http://ibdws.sdsu.edu/~ibdws/); genes: *COI* cytochrome oxidase I, *cytb* cytochrome *b*; *RH* rhodopsin, *RAG1* recombination activating gene 1

## Results

### Samples and datasets

In contrast to COI, fresh material for amplification or Genbank sequences were not available for cytb and two nuclear genes for all putative species of *Phoxinus* in Europe. Even though several museums were contacted in order to obtain this material, some of the investigated species are still missing from the cytb and nuclear dataset. Material collected by (or donated to) our group includes *P. apollonicus* (clade 7), *P. karsticus* (clade 7), *P. ketmaieri* (clade 1), *P. likai* (clade 1), *P. lumaireul* (clade 1), *P. phoxinus* (clade 10), *P. septimanae* (clade 11), *P. csikii* (clade 5) and *P. marsilii* (clade 9). Additionally, *Phoxinus* sp. 1–5 (clades 2, 3, 4, 6 and 8) are also presented. For rhodopsin (but not for RAG1), sequences representing *P. bigerri* (clade 13), *P. colchicus* (clade 14), *P. strandjae* (clade 14) and *Phoxinus* sp. 7 (clade 17) were available in the Genbank. In Genbank, one cytb sequence was present for *P. bigerri* (clade 13) and one for *Phoxinus* sp. 7 (clade 17). Material for *P. morella*, *P. strymonicus* and *Phoxinus* sp. 6 (clade 16) was not available.

In total 559 651-bp-long sequences of COI were used, of which 322 were new and 241 were downloaded from Genbank. They collapsed to 141 unique haplotypes. For cytb, 385 1091-bp-long sequences were used, of which 48 were new with the others originating from previous studies. The sequences collapsed to 214 unique haplotypes. For rhodopsin, 85 (871 bp long) randomly chosen samples representing available clades/species were successfully amplified, while fourteen sequences (only 782 bp long) were downloaded from Genbank. RAG1 amplification resulted in 100 (1413 bp long) sequences.

All sequences are available under the Genbank accession numbers MF407678 - MF408232 .

### Museum material

Information on successfully amplified museum material is reported in Additional file 3: Table S6. From the type material of *P. marsilii*, only one of the six specimens amplified successfully for the complete COI region and 473 bp for cytb, while of the six specimens collected close to the type locality of *P. csikii*, all amplified successfully for the complete COI and two of them also for 473 bp of cytb. Museum specimens from Stepenitz River near to Upahl, Germany, collected in 1981 (ZMB 31261_1 and _2) amplified successfully for the first two parts of COI (C1 + C2, 391 bp). Amplification of (partial) nuclear genes from museum material was unsuccessful.

### Mitochondrial DNA

#### Phylogenetic analysis

**Fig. 2:**
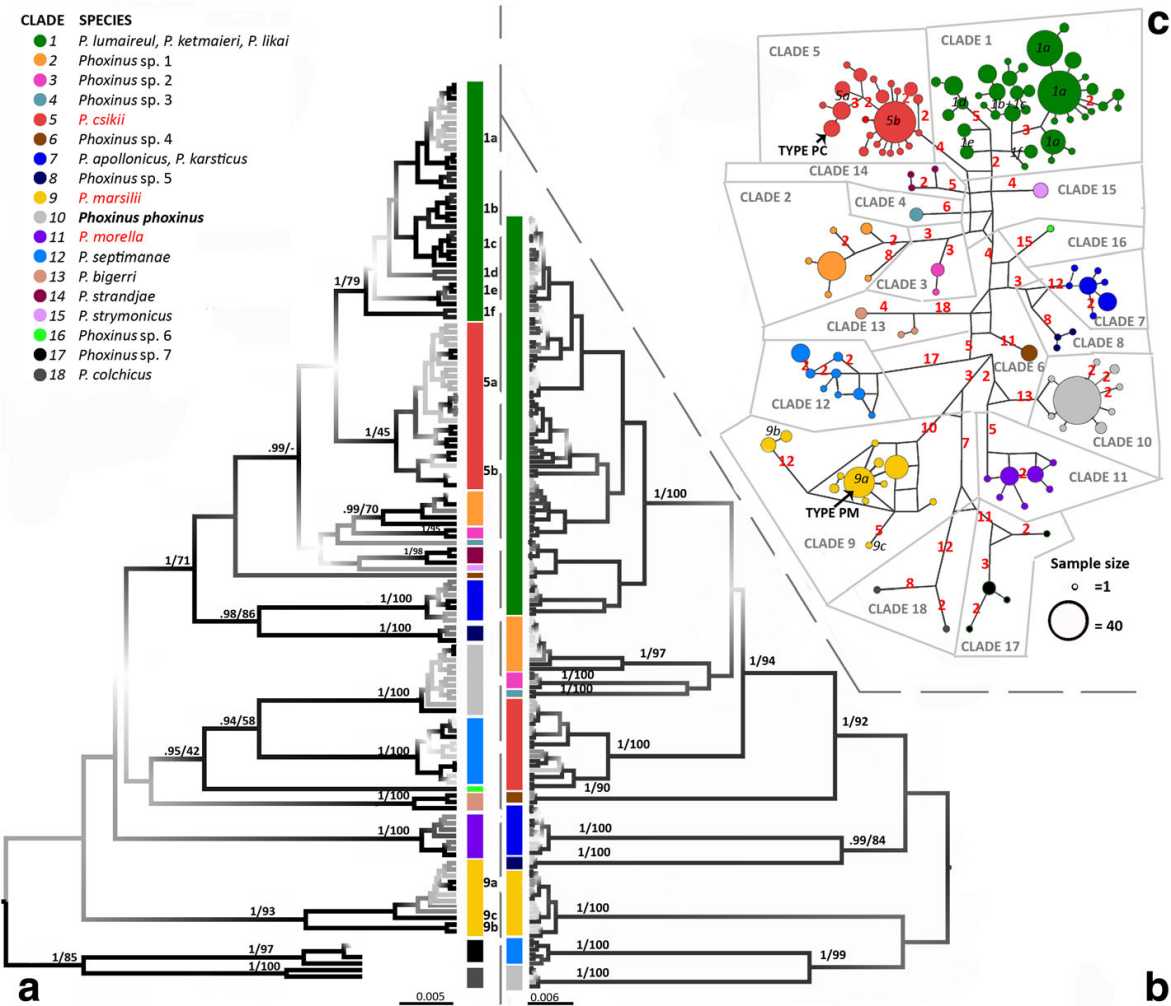
Phylogenetic reconstruction and haplotype network for revision of the genus *Phoxinus*. Figure 2**a** Phylogenetic tree constructed from the barcoding region of mitochondrial gene cytochrome oxidase I (COI). Collapsed alignment includes 139 unique haplotypes. The tree was created using Bayesian inference (BI) with BEAST 1.8.0 [26]. Branches carry posterior probabilities (PP) and bootstraps (BS) from the tree constructed with the Maximum-Likelihood (ML) method (PhyML; [29]). Weakly supported nodes are *grey* and only PP above 0.9 are shown for the main clades (no subclades). -, denotes lack of bootstraps originating from the difference between the BEAST and ML trees. Figure 2**b** Phylogenetic tree constructed with two partitions: COI and cytochrome *b*. Collapsed alignment includes 162 unique haplotypes. As in Fig. 2**a**, BI and ML were used. Because PhyML does not support partitions, GARLI v.2.01 [30, 31] was used for ML. Figure 2**c** An unrooted minimum-spanning network was constructed with COI using the median joining algorithm [32] implemented in Network 5.0 (www.fluxus-engineering.com) with default settings. The position of the type material in the network is denoted with TYPE PM for *Phoxinus marsilii* and TYPE PC for *P. csikii*

**Fig. 3:**
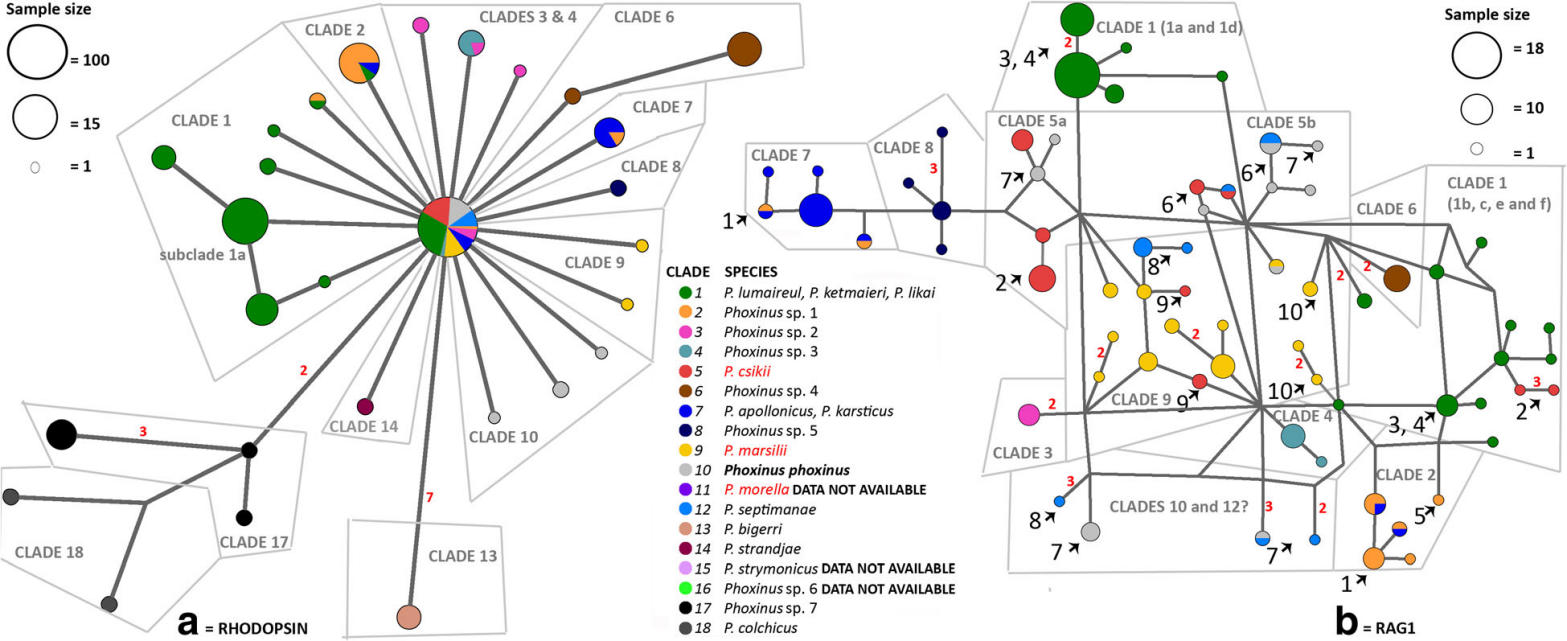
Haplotype networks constructed with nuclear DNA. The colours represent lineages detected by mitochondrial DNA analysis. For both genes, the gametic phase of heterozygous individuals was determined using Phase 2.1 [38, 39], then an unrooted minimum-spanning networks were constructed with median-joining algorithm [32] implemented in Network 5.1 (www.fluxus-engineering.com) with default settings. Figure 3**a** Rhodopsin haplotype network was constructed from 782 base pair long phased alignment. Figure 3**b** Recombination activating gene 1 (RAG1) haplotype network was constructed using 1413 base pair long phased alignment. The *arrows* denote areas, where hybrids were detected and correspond to Fig. 1

Phylogenetic analysis of the COI dataset detected 18 clades, denoted by colours in Figs. 1, 2 and 3. Of the 18 detected clades, six corresponded to one of the currently valid *Phoxinus* species, while two of the clades combined more than one species (clade 1 – P. *lumaireul*, *P. ketmaieri*, *P. likai* and clade 7 – *P. apollonicus*, *P. karsticus*). Seven of the clades have not yet been formally assigned (*Phoxinus* sp. 1–7) and three clades correspond to the species, which were until now considered synonyms of *P. phoxinus* – *P. csikii*, *P. marsilii* and *P. morella*. Whereas individual clades in the COI tree were well supported, the relationship among them was unclear. In general, good support for the clades, but very weak support for the deeper nodes, was a common feature of the phylogenetic reconstruction of all four datasets. The common pattern also included good support for a shared origin of clades 1–5, even though this topology was not always supported in ML analysis. In addition, the relationship of clades 7 and 8 as sister groups was well supported in all datasets. In some of the clades (e.g., subclades 1a–f, 5a, 5b, 9a, 9b; Fig. 2a) sub-structures were also present. In Fig. 2b, the dataset combining two partitions—1742-bp-long complete COI and complete cytb regions—is shown, pointing to a common origin of clades 1–6. Another two datasets—cytb and COI + cytb partial—are reported in the supplementary material (Additional file 3: Figures S1 and S2).

Genetic distances among subclades 1a–f based on COI were all 1%, except between clade 1d and clades 1a–1c, at 2%. The genetic distance between 5a and 5b was zero and between 9a and 9b, 2%. In addition, some genetic distances between the different clades were in the same range: for example 1% between clade 15 and 1e and 1f. The largest genetic distances were between clades 17 and 1a, 17 and 1c, 17 and 7, 17 and 13, and between 18 and 7, at 7%. Genetic distances based on cytb were larger. Pairwise genetic differences between clades are reported in the supplementary material (Additional file 3: Tables S8–S11).

The haplotype network showed good separation of clades 6–17, with more than 20 mutational steps separating them from each other. However, clades 1–5, 14 and 15 were not as well separated (Fig. 2c), with eight mutations between some samples from clades 1 and 5. Similarly, there were eight mutations between clades 1 and 15.

Successfully amplified type material of *P. marsilii* clustered within clade 9a on the phylogenetic tree and exhibited the same haplotype as freshly collected material from Vienna, Austria. Museum material collected from close proximity to the *P. csikii* type locality clustered within clade 5b, but exhibited a unique haplotype. The genetic distance (based on COI) between clades 10 (*P. phoxinus*) and 9 (*P. marsilii*) was 5%, while that between clades 10 and 5b (*P. csikii*) was 7%. The distance between clades 10 and 1 (*P. lumaireul*) ranged from 6 to 8%, depending upon the subclade. In the network, the type of *P. marsilii* was one of the central and most abundant haplotypes (marked as type PM in Fig. 2c). The six samples collected near the *P. csikii* type locality formed their own haplogroup (marked as type PC). Museum material from northern Germany (ZBM-31261) clustered within clade 11. The two samples exhibited the same haplotype as that from Prepere (Elba drainage, Czech Republic).

#### Species delimitation

Using ABGD, 25 species were detected in the COI dataset. In addition to the 18 clades, some subclades were denoted as separate species, namely 1a, 1b + 1c + 1e + 1f and 1d. Clade 9 separated into three species represented as subclades 9a and 9b and one haplotype denoted 9c (one of the specimens collected from Beskydy, Oder drainage, Czech Republic). Finally, clade 17 separated into two species, one being the most external haplotype (Volga River, Russia) and the remaining two the other species. Both samples in clade 18 separated as two distinct species.

The GMYC method gave very similar results to the ABGD method, except that it divided some clades even further. The previously detected species 1b + 1c + 1e + 1f was divided further into two species 1b + 1c and 1e + 1 f. Also, subclades 5a and 5b were identified as separate species. The PTP method gave similar results, apart from uniting 1a + 1b + 1c + 1e + 1f as a single species, and re-uniting the separated haplotype 9c with clade 9a. However, this method split clade 13 into two species. The results are reported in more detail in Additional file 3: Table S12.

#### Isolation by distance

The correlation between genetic and geographic distance suggested in clade 1 (Z = 21.5051, *r* = 0.1152) was not supported statistically (*p* = 0.1080), while isolation by distance at least partially explained the structure of clades 5 (Z = 8.8396, *r* = 0.7461, *p* < 0.0001) and 9 (Z = 2.6242, *r* = 0.7531, *p* = 0.0477).

### Nuclear DNA

#### Haplotype networks

After the gametic phase of the sequenced samples was inferred, the run with the highest probabilities was chosen for further analysis. For rhodopsin, only one (of the 85 sequences) was omitted from further analysis due to low statistical support. Determining gametic phase was less successful with RAG1, as a number of resolved haplotypes exhibited low probability scores for several single nucleotide polymorphisms. Consequently, 19 samples (of 101) with more than one SNP exhibiting probability under 0.9 were omitted from further analysis.

Rhodopsin sequences produced in this study (871 bp) exhibited only 18 polymorphic sites, while the combined dataset with sequences from the Genbank (782 bp) displayed 36 polymorphic sites. In the combined dataset, 89 bp were jointly deleted from both ends of the alignment, however no polymorphic sites were removed. Thus, only one haplotype network was constructed using a 782 bp long segment. The network showed the conservative nature of rhodopsin, with most of the samples exhibiting the same, central haplotype (Fig. 3a). Clades 5 (*P. csikii*) and 12 (*P. septimanae*) are only represented by the central haplotypes, while clades 1 (*P. lumaireul*), 2 (*Phoxinus* sp. 1), 3 (*Phoxinus* sp*.* 2), 4 (*Phoxinus* sp*.* 3), 7 (*P. karsticus*), 9 (*P. marsilii*) and 10 (*P. phoxinus*) also exhibit some unique haplotypes. However, these are distinct only by one mutation and are spreading out separately from the centre (i.e. they are not interconnected with each other, Fig. 3a). The exception is clade 1 (*P. lumaireul*), where a group of haplotypes, represented mostly in samples from subclade 1a are showing a more complex structure. Clades 6 (Croatian Krka samples - *Phoxinus* sp*.* 4), 8 (Ohrid (FRY Macedonia) samples - *Phoxinus* sp*.* 5) and 14 (*P. strandjae*) are also represented by haplotypes distinguished by only one mutation, but they have no haplotypes identical to the central one. Clade 17 (*Phoxinus* sp*.* 7) and 18 (*P. colchicus*) form a separate group, in which first haplotypes of the clade 17 branch off (two mutations difference), and from those two haplotypes of the clade 18 are separated (one mutation). In the clade 17, Baltic samples form a group separated from the samples from Russia by three mutations. The most diversified are haplotypes of the clade 13 (*P. bigerri*), which are separated from the central haplotype by seven mutations. Data for clades 11 (*P. morella*), 15 (*P. strymonicus*) and 16 (*Phoxinus* sp*.* 6) was not available.

RAG1 sequences displayed 59 polymorphic sides, three of which had more than two variants. The network is interconnected, and displays many theoretical intermediate states (Fig. 3b). The differences between the haplotypes are mostly represented by one-mutational steps. None of the clades is well separated from each other, except possibly clades 3 (*Phoxinus* sp. *2*) and 6 (*Phoxinus* sp. 4). Clades 2 (*Phoxinus* sp. 1), 4 (*Phoxinus* sp. 3), 7 (*P. karsticus*), 8 (*Phoxinus* sp. 5) and subclades 1a (*P. lumaireul* sensu stricto) and 5a (*P. csikii* sensu stricto) can also be recognized. The centre of the network possibly represents clade 9 (*P. marsilii*), but the pattern is distorted by a number of hybrids with (sub) clades 5b (arrow no. 9) and 12 (arrow no. 8). Further, hybrids and/or incomplete lineage sorting can be recognized between clades 2 (*Phoxinus* sp. 1) and 7 (*P. karsticus*; arrow 1), subclades 1f and 5a (arrow 2), subclades 1a and 1b (arrows 3 and 4), clade 2 and subclade 1b (arrow 5), subclade 5b and 12 (arrow 6), clades and subclades 5a, 5b, 10 and 11 (arrow 7). At the bottom of the network, there are some haplotypes, which are separated with two or even three mutational steps and could possibly represent clades 10 (*P. phoxinus*) and 12 (*P. septimanae*). Notably, clade 1 is separated in two groups, one is mostly represented by haplotypes exhibited by samples from clade 1a (but also 1d), while the other includes haplotypes represented in clades 1b, 1c, 1e, 1 f. Between these two groups, hybrids were also detected. Data for clades 11 (*P. morella*), 13 (*P. bigerri*), 14 (*P. strandjae*), 15 (*P. strymonicus*), 16 (*Phoxinus* sp. 6), 17 (*Phoxinus* sp. 7) and 18 (*P. colchicus*) was not available.

## Discussion

### Revising species complexes

With the rapid growth in the number and scale of molecular phylogenetic studies and barcoding projects it has become increasingly clear that levels of biodiversity are highly underestimated, as a result of cryptic diversity. Nevertheless, the discovery of cryptic lineages is, because of difficulties with species identification by molecular analysis [43, 44], often not followed up with formal species description [45]. Thus, a huge amount of species richness probably goes without formalization and remains unprotected by conservational efforts. To aid in such formalization of species detected in barcoding projects, Puillandre et al. [9] proposed a workflow for species delineation. First, barcoding data are analysed with species delimitation programs, such as ABGD and GMYC, to form a primary species hypothesis. Second, additional molecular markers, morphological or ecological data, or both, are used to confirm the primary as a secondary species hypothesis. In the present study, a converse approach was tested on an example of the *Phoxinus* species complex in Europe. Species described in recent and historical morphological studies were treated as the primary species hypothesis. However, morphological characters used for species delimitation proved to be unreliable. For example, Kottelat and Freyhof [12] used body measurement ratios to discriminate between putative species; yet it has been shown by geometric morphometric studies that some of these ratios are dependent upon the environment [15, 16]. Additionally, Knebelsberger et al. [17] found four different lineages populating the area of the *P. phoxinus* type locality that became obvious to the authors only after molecular analysis. Bianco and De Bonis [19] based three out of four species descriptions on only one or two populations per species, represented by 5–12 specimens, excluding possible variability range of the characters used. Therefore, morphologically defined species were evaluated with molecular data to form secondary species hypotheses. Of the fourteen primary species hypotheses, analysis based on mtDNA rejected three of the species; three required further analysis and eight were supported as secondary species hypotheses. Nuclear DNA analysis corroborated the rejection of the two of the species previously excluded by the mtDNA analysis. Further, of the eight well supported mtDNA species, two of the species were unequivocally corroborated by nuclear DNA analysis. Finally, for six additional species, nuclear DNA offered limited support (Table 1, discussed also below), and thus the approach of conversing morphological with molecular data to form secondary species hypotheses has proven to be a useful tool for revision of species complexes.

However, as previously pointed out in the Background [5, 7, 8], the use of molecular data in species delimitation is not without limitations. Especially in fishes, where numerous hybridization events and mitochondrial captures were reported (e.g. [46–48]), discrepancies between gene trees (most prominently between mitochondrial and nuclear genes) have been detected [49]. Further, while mtDNA with its simpler mode of inheritance, differences in effective population size and higher mutation rates offers well separated (and well supported) clades, it is hard to find a single nuclear marker with enough resolution to delimit closely related species (e.g. [23]. Correspondingly, using two nuclear genes, rhodopsin and RAG1, did not sufficiently clarify the status of all of the species within the genus *Phoxinus* analysed in this study. As previously shown [23], rhodopsin has proven to be too conservative, and was able to unequivocally confirm only the two most geographically distant species in *Phoxinus* – *P. bigerri* and *P. colchicus*. RAG1 was also previously shown to be insufficient for species delimitation [18] and even though longer fragment was used in this study, which showed to be more promising (Fig. 3b vs. Additional file 3: Figure S3 in the supplementary data), support for species identified by morphological and mtDNA data remained limited. The lack of phylogenetic signal as detected in this study is according to Funk and Omland [7], one of three possible reasons for discrepancies between mitochondrial and nuclear trees. Two other reasons are incomplete lineage sorting and (ancient or recent) introgressive hybridisation, which were likewise detected herein. Hybrids between clades 7 (*P. karsticus*) and 2 (*Phoxinus* sp*.* 1) were reported previously [18] and explained as ongoing contact between populations through underground water connections in that area (Bosnia-Herzegovina; marked by arrow 1 in Fig. 1 and 3b). Additional regions, where a similar phenomenon was suggested, are in south-east Serbia (between clade 5 and subclade 1f), in Croatia (between the subclades 1a and 1b) and in Slovenia (also subclades 1a and 1b), corroborated in this study by both mixed mitochondrial and nuclear haplotypes (marked with arrows 2–4). Newly observed was hybridisation between clades 1 and 2 (Bosnia-Herzegovina; arrow 5). However; based on only four loci it is hard to distinguish, whether the detected pattern is a consequence of incomplete lineage sorting or introgressive hybridization [50], especially because most of the occurrences were detected in the contact zones. Though, the clades 2 and 7 seem well divided (on the opposite sides of the RAG1 network, Fig. 3b), thus (secondary) hybridisation of two separated lineages is more plausible.

In comparison to the Balkan area, the situation in the central Europe is even more challenging. As expected from previous studies [17] and further expanded by mtDNA analysis herein, hybrids were present in Lake Geneva, Switzerland (probably originally populated by *P. septimanae*, clade 12, arrow 6) and Agger River, Germany (at least four different lineages, arrow 7), resulting in very complicated RAG1 network. Additionally, hybrids were present in the introduced population in Italy (personal communication with G. B. Delmastro; marked with arrow 8), and Austria (arrow 9), exhibiting close similarity to clade 9 - *P. marsilli*. In the Italian population, hybrids between *P. lumaireul* (clade 1) and the nearby clade 12 (*P. septimanae*) would be expected, but as the population is not natural, several populations from different watersheds might have been introduced. The hybrids in the Austrian population possibly occur naturally, as they are in the contact zone between clades 5 and 9 (*P. csikii* and *P. marsilli*). Finally, there seem to be some incomplete lineage sorting between the clade 9 and subclades 1b–c (arrow 10 in Fig. 3b). If the lack of signal would be the only reason for limited delimitation properties of rhodopsin and RAG1, additional nuclear genes might help to resolve the relationships in *Phoxinus* (as for example in [51, 52]). However, because of the numerous natural or human introduced [17, 53, 54] hybridisation events, finding intact populations is crucial for resolving *Phoxinus* phylogeny. Using museum material (collected before massive stocking) for species delimitation might be another option, and while amplification of several nuclear markers from museum material is possible, it is extremely labour-intensive [55] and the problem of insufficient phylogenetic signal in reduced number of nuclear markers remains. Thus, new approaches such as high throughput genotyping, which have proven very useful to delimit species in hybrid zones [56], seem most promising, and steps have been made towards extension of the barcoding concept with genomic data [57]. Besides, there have been advances in combining high-throughput DNA sequencing with museum specimens [58]. Finally, the utility of morphological characters for species delimitation in *Phoxinus* should not be excluded, for example, Ramler et al. [16] suggested the inclusion of all body planes for finding morphologically distinguishing features. However, the problem of hybridisation events in *Phoxinus* possibly extends to morphology and could be the reason why several studies have not been able to find stable characters for species delimitation [12, 13, 19]. Thus, in species complexes such as *Phoxinus*, with closely related species, limited morphological information and numerous hybrid zones, only most modern approaches combined with integrative taxonomy will possibly enable species delimitation.

### Revision of European *Phoxinus*

The results of the revision of European *Phoxinus* are reported in Table 1. Of eleven species proposed by morphological studies, two—*P. bigerri* (clade 13) and *P. colchicus* (clade 18)—are confirmed with molecular data as secondary species hypotheses. *P. karsticus* (clade 7) is also well separated as clade in both mtDNA analysis and RAG1 network. If considering only subclades containing their type localities, additional two species—*P. lumaireul* (subclade 1a) and *P. csikii* (subclade 5a)—are corroborated with mtDNA and nuDNA analysis. *P. marsilii* (clade 9) is strongly supported by mtDNA analysis, while nuDNA analysis offers only limited support. For three species—*P. ketmaieri* (subclade 1a), *P. likai* (subclade 1a) and *P. apollonicus* (clade 7)—synonymization is proposed. The status of three species—*P. strandjae*, *P. strymonicus, P. morella*—remains uncertain and additional sampling is needed.

#### *P. bigerri* (clade 13) and *P. colchicus* (clade 18)

The two best supported species by both mtDNA and nuDNA data are *P. bigerri*—clade 13 and *P. colchicus*—clade 18. And even though they are not represented in the RAG1 network, they are well divided on the basis of the conservative rhodopsin gene. According to the species delimitation programs, there might be even more than one species within each of the clades, in which case, *P. bigerri* would be attributed to a subclade with samples collected in Bonnemazon, France (20 km east of the type locality - River Adour in Tarbes) and *P. colchicus* to Natanebi drainage, Black Sea basin. Nevertheless failure to sample intermediate haplotypes could also be the reason causing the over-splitting in the clades 13 and 18, thus further sampling is needed.

#### *P. karsticus* (clade 7) and *P. apolonicus* (clade 7)

Bianco & De Bonis [19] described *P. karsticus* from Trebišnjica River (Donja Kočela, Bosina-Herzegovina in Additional file 2: Table S2; a sinking river that flows underground to the Adriatic Sea) and *P. apollonicus* from Morača River (Duga, Montenegro in Additional file 2: Table S2; Skadar Lake basin, Adriatic Sea basin). In the present study, the analysis of the samples from both locations showed that the intra-population genetic distance is larger than the inter-population distance between these two sampling sites (based on COI; data not shown). Morača and Trebišnjica Rivers share the same haplotypes also based on nuDNA (rhodopsin and RAG1), thus there is support for one, but not for both species. Acting as First Reviser (Art. 24.2.1. of ICZN), we synonymize the simultaneously published names *P. apollonicus* and *P. karsticus*, and give precedence to the name *P. karsticus* for clade 7, with the distribution range of Skadar Lake basin and some surrounding sinking streams.

#### *P. phoxinus* (clade 10) and *P. septimanae* (clade 12)

Clade 10—*P. phoxinus* and clade 12—*P. septimanae* are well supported mitochondrial lineages, which are unequivocally recognized by species delimitation programs as separate species. However, in the rhodopsin network, *P. septimanae* is only represented by the most abundant central haplotype, and while *P. phoxinus* samples display a few unique haplotypes, no separated network-forming structure was detected. In the bottom of the RAG1 network, some distant haplotypes are represented, separated by more than three mutational steps from their closest neighbours, which could represent clades 10 and 12. Yet, all the samples attained in this study, which were according to mtDNA classified in clades 10 or 12, come from introduced and highly mixed populations (Agger River, Germany; Lake Geneva, Switzerland; Ceresole Lake, Italy) thus more sampling will be needed to draw firmer conclusions.

#### *P. lumaireul* (clade 1)

There is no doubt that *P. lumaireul* is genetically distinct from *P. phoxinus* (genetic distance based on COI was 8%, while the maximum distance in our dataset was 9%), supporting Kottelat’s [13] revalidation of this species and rejecting concerns raised by Bianco [14] and Bianco & De Bonis [19]. However, the species range of *P. lumaireul* is still debatable, because within clade 1 up to six subclades were detected based on mtDNA. Subsequently, the relationship between geographic and genetic distance was tested to evaluate whether the subclades evolved as a consequence of isolation by distance, but showed that IBD does not seem to play a role in the structure of clade 1. Thus, *P. lumaireul* corresponds to Adriatic subclade 1a (including the type locality - Po drainage, Italy), which is also supported by nuclear data. Even though the genetic distance (1–2%) and a small number of mutational steps between the subclades (haplotype network, Fig. 2c) based on mtDNA point to the common origin of the clade 1, subclade 1a can be recognized in the rhodopsin network, while the haplotypes belonging to subclades 1b-1f are mostly identical to the central haplotype. In RAG1, clade 1 is separated in two groups, one mostly represented by subclades 1a and interestingly 1d, and second with the rest of the subclades. This separation of the subclade 1a is also in congruence with geography, because of its Adriatic origin, while other subclades belong to the Danube watershed. However, the common haplotypes shared between 1a and 1d in the RAG1 network (which geographically are not adjacent) is hard to explain. In a preliminary study [42], species delimitation of 1a from 1b–c based on morphological characters proved to be challenging, pointing again to a common origin of the clade 1; however until more morphological and molecular data is gathered, *P. lumaireul* is restricted to the subclade 1a with the species range in the North Adriatic Basin in Italy, Slovenia and Croatia. (For detailed distribution areas of subclades 1a-1f see also [18]).

#### *P. ketmaieri* (clade 1) and *P. likai* (clade 1)

In 2015, Bianco & De Bonis [19] described *P. ketmaieri* from Krk Island, however the genetic distance between the Krk samples (Baška, Croatia, Additional file 2: Table S2) and the rest of clade 1a (*P. lumaireul*) collected in this study is very low (0.6% based on COI; data not shown). In the rhodopsin network, Krk samples display unique haplotype as a part of subclade 1a sub-network, while in RAG1 network, they exhibit the same haplotype as many other samples (the biggest circle in the clade 1a sub-network). The *Phoxinus* samples from Zrmanja River (Mokro Polje, Croatia, Table S2), which were according to Bianco & De Bonis [19] also assigned to *P. ketmaieri*, were found to belong genetically to two subclades, 1a and 1b. The hybrids are confirmed by mtDNA and nuDNA analysis. Thus, because of lack of genetic differentiation on one hand and possible hybridisation not detected by Bianco & De Bonis [19] on the other hand, *P. ketmaieri* should be synonymized with *P. lumaireul*.

*Phoxinus likai* from Otuča River near Gračac, Croatia (erroneously spelled Oruča and placed in Bosnia and Herzegovina in Bianco & De Bonis [19]) was not analysed though we obtained samples from Lovinac River (Gračac, Table S2) whose spring is about 1 km from Otuča River. These samples cluster in subclade 1b, which is according to mtDNA closely related to the subclade 1a (but see also discussion about clade 1 and nuclear markers), thus synonymization with *P. lumaireul* is suggested. However, the samples from Lovinac are only represented by cytb, so further investigation is necessary to resolve the status of this species.

#### *P. marsilii* (clade 9)

Based on mtDNA, clade 9—*P. marsilii*—is well differentiated from clade 10 (*P. phoxinus*), as well as from all other surrounding clades, supported by phylogenetic trees, COI haplotype network and genetic distance calculations. In addition, Mantel test found a positive correlation between the subclades 9a, 9b and 9c, showing that the split between them is a consequence of isolation by distance. However, based on nuDNA networks, the support for *P. marsilii* is limited. There are some unique haplotypes present in the rhodopsin network and a central clade can be recognized in the RAG1 network, but the pattern is distorted by hybrids between the clades 9 and 5b (*P. csikii*), and limited separation of the clade 9 from (sub) clades 1a, 1b, 1c, 5b and 4. Nevertheless, *P. marsilii* was re-established as a valid species, also because in case insufficient delimitation will be presented in further studies, and surrounding clades 1 (*P. lumaireul*) and 5 (*P. csikii*) will be merged under the same name, the material from this area (Vienna) was described first (see Table 1). Thus, the name *P. marsilii* has priority according to Art. 23 of the International Code of Zoological Nomenclature (IUZN). The distribution range of *P. marsilii* is determined as the middle and lower Danube drainage, mostly the left tributaries (Fig. 1). It was also detected in Oder and Elba drainages, Czech Republic (Additional file 2: Table S2), though this could be a consequence of human introductions as detected elsewhere [17, 53, 54].

#### *P. csikii* (clade 5)

While based on mtDNA clade 5—*P. csikii*—is well separated from *P. phoxinus* (clade 10), *P. marsilii* (clade 9), and *P. septimanae* (clade 12), is the genetic distance dividing clade 5 and adjacent clade 1 (*P. lumaireul*) (only) between two and 3%. In contrast, all three species delimitation programs unequivocally separated the two clades, and the genetic distances based on cytb and the phylogenetic reconstruction based on COI + cytb (Fig. 2b) show a more pronounced distinction between the clades. In addition, Ramler et al. [16] found morphological differences—deeper bodies as well as deeper and shorter caudal peduncles—between some of the populations of clades 1 and 5 that seem to be unrelated to the habitat. Regarding nuDNA, clade 5 is only represented in the rhodopsin network by a central haplotype. However, in the RAG1 network; there seem to be support for two subclades 5a and 5b (Fig. 3b). In the Fig. 3b, the colours are denoted according to mtDNA lineages; and in all the sampling sites, from which the samples classified into encircled clades 5a and 5b, hybrids with the clade 5 were expected (Agger River, Lake Geneva). As there seem to be sufficient support for the subclade 5a (which includes locality of the neotype - Rožaje, Montenegro) the subclade was revalidated as *P. csikii* with a distribution in the central Balkan Danube drainage (Fig. 1). Regardless of positive IBD-correlation between the subclades 5a and 5b, further studies are needed to determine the origin of the subclade 5b.

#### *P. strandjae* (clade 14) and *P. strymonicus* (clade 15)

Similarly, as within some subclades, genetic distances based on COI between clades 14 and 15 and clades 1–5 are short (see also haplotype network, Fig. 2c), the extreme being 1% difference between clades 1e + 1f and 15. In the rhodopsin network there is some limited support for clade 14 and the species delimitation programs support them as separated species, namely *P. strandjae* (clade 14) from Turkey (Sapanca drainage, Black Sea basin) and *P. strymonicus* (clade 15) from Greece (Strymonas drainage, Aegean Sea basin). However, further studies and denser sampling are needed to resolve the status of these two species in relation to *P. lumaireul*, *P. csikii* and other Balkan *Phoxinus* (clades 2–4).

#### *P. morella* (clade 11)

A well supported clade 11 is spreading from Czech Republic through Germany towards the Baltic Sea and seems to be well separated from the neighbouring clades 10 (*P. phoxinus*), 5b and 9 (*P. marsilii*). However; the species delimitation was performed based on mtDNA, thus further sampling (including at the type locality) and amplification of nuclear genes is needed to determine the status of this species.

### Unassigned clades

Regardless of the allocation of two (possibly three) available species names to detected genetic lineages, seven clades remain without a name available. According to the criteria mentioned above [9], these lineages, detected with analysis of COI with species delimitation programs, can be considered as primary species hypotheses. However, the sampling density ought to be increased as it was not equally distributed across the clades and putative species, possibly causing over-splitting by species delimitation programs [59]. Nevertheless; clades 4 and 17 seem to be well supported by both mtDNA and nuDNA and are potential candidates for new species.

## Conclusion

In the present research, contrasting controversial morphological species descriptions against molecular data have proven to be a useful approach to revision of species complexes. The current recognized species of the European *Phoxinus* complex has been revised, offering a new overview of European *Phoxinus* and providing a solid foundation for further studies.

### Taxonomical implications

#### Designation of a lectotype of *Phoxinus marsilii*

According to ICZN (Art. 74.1, 74.7) a lectotype is herein designated to become the unique bearer of the name of *P. marsilii*. It is properly labelled in the NMW and can be identified by its morphological features described below.

In 1836, two specimens were taken into the collection of the Hof-Naturalien-Cabinett, the forerunner of the NMW, as *Phoxinus marsilii* (Acqu. Nr. 1836.I.20). However, the NMW-51225 sample with this acquisition number contains six specimens. The number and sizes of the specimens Heckel [60] used to base his description of *P. marsilii* upon is unclear, though it is obvious from the original description that more than one was used. We consider all six specimens as syntypes and designate specimen NMW 51225:2 (Additional file 3: Figure S4) as the lectotype of *P. marsilii* Heckel, 1836.

For the type locality Heckel described *P. marsilii* from clear brooks of the environs of Vienna and beyond (“… in allen klaren Bächen der Wien-Gegend und weiter …”).

The lectotype is characterized by lateral line extending close to caudal fin base (87 scales in lateral series: 74 pored and 13 non-pored); two patches of breast scales, not separated by scaleless area (three rows of scales, 4–6th, confluent); no scales between pelvic and pectoral fins; 8 branched rays in both dorsal and anal fins (last two rays originating on single pterygiophore); 16/16 branched pectoral fin rays; 7/7 branched pelvic fin rays; total vertebrae, 40 (22 abdominal, and 18 caudal); depth of caudal peduncle, 9.8% standard length (SL), 35% caudal peduncle length and 60.7 % body depth; body depth, 16.1% SL.

The Senckenberg Museum in Frankfurt am Main, Germany (SMF) holds two specimens as syntypes of *P. marsilii* (SMF 1980), received in 1844 from the NMW. From the NMW acquisition sheet for that year it is evident that two specimens labelled *Phoxinus marsilii* Heck. were sent to Prof. Joh. Müller but had been sampled in northern Italy, in brooks at Treviso (Acquisition Nr. 1844.III.3), not in the surrounds of Vienna. As such, they are not syntypes of *P. marsilii*.

For the vernacular name, we propose the name Viennese minnow (German “Wiener Elritze”).

#### Designation of a neotype of *Phoxinus csikii* and its type locality

*Phoxinus csikii* was described from a karstic brook near Korita (43°00′25″N, 19°58′03″ E), Bijelo Polje region in northern Montenegro [61]. The brook is a sinking stream at the border of the Lim (Drina–Sava–Danube) and Ibar (Zapadna Morava–Danube) drainages. The two syntypes, one juvenile (46 mm total length, TL) and one adult female (75 mm TL), of *P. csikii* were deposited at the Hungarian Natural History Museum (MNSB) in Budapest. The original type series is lost (see below). Because several *Phoxinus* species occur in the Danube region (see below), there is an explicit need for the designation of a neotype (Art. 75.3. of ICZN).

We designate the specimen NMW-51266, 89.5 mm SL (Additional file 3: Figure S5) as the neotype of *Phoxinus csikii*. All qualifying conditions (Art. 75.3 of ICZN) are met: the neotype is designated to clarify the taxonomic status of the species (Art. 75.3.1), and the original description provides a sufficiently full differentiating description of a larger syntype (Art. 75.3.2). The two syntypes of *P. csikii* were donated to MNSB in July 1917 by Ernst (Ernő) Csiki, a Hungarian entomologist and director of the museum at that time. Dr. Judit Vörös, the curator of the fish collection in this museum informed that, at present, these specimens are absent from the collection as having been destroyed, probably by a fire in 1956 (Art. 75.3.4.).

The neotype was collected close to the original type locality (Art. 75.3.6.) at Rožaje, Montenegro [Rozaj] (42°50′39″N, 20°10′00″E), on the Ibar River, tributary to the Zapadna Morava river, a tributary of the Danube. The neotype is consistent with the original description (Art. 75.3.5) and can be unambiguously recognised (Art. 75.3.3.) through having the following characters: incomplete lateral line almost continuous to origin of anal fin with few single pored scales on caudal peduncle (last pored scale in middle of caudal peduncle); 90 scales in lateral series (51 pored, 39 non-pored); two patches of breast scales separated distinctly by scaleless area; posterior one-third of area between pectoral and pelvic origins scaled; eight branched rays in both dorsal and anal fins (last two rays originating on single pterygiophore); 16/15 branched pectoral fin rays; 7/7 branched pelvic fin rays; total vertebrae, 41 (22 abdominal and 19 caudal); depth of caudal peduncle, 10.3% SL, 40.3% caudal peduncle length and 43% body depth; body depth, 24.8% SL.

## Additional files

Additional file 1: Table S1. Characters used in the literature for distinguishing species/subspecies of P. phoxinus s.l. (XLS 39 kb)

Additional file 2: Table S2. Sampling sites used in the study, with corresponding drainage, basin and reference, where applicable. (XLSX 111 kb)

Additional file 3: Table S3 – S12 and Figures S1–S5. ᅟ(DOCX 2760 kb)

## Abbreviations

ABGD: Authomatic Barcode Gap Discovery
Bp: Base pair
COI: Cytochrome oxidase I
Cytb: Cytochrome b
GMYC: General Mixed Yule Coalescent model
IBD: Isolation by distance
iBOL: International Barcode of Life (project)
IUZN: International Code of Zoological Nomenclature
mtDNA: Mitochondrial DNA
NMW: Catalogue numbers of the Natural History Museum Vienna
nuDNA: Nuclear DNA
PCR: Polymerase chain reaction
PTP: Poisson Tree Processes
RAG1: Recombination activating gene 1
ZMB: Catalogue numbers of the Museum of Natural History Berlin
