# Corrigendum to «Contrasting morphology with molecular data: an approach to revision of species complexes based on the example of European (Cyprinidae)» by Palandačić et al. 2017

**DOI:** 10.3897/BDJ.5.e21772

**Published:** 2017-10-24

**Authors:** Anja Palandacic, Alexander M. Naseka, David Ramler, Harald Ahnelt

**Affiliations:** 1 Naturhistorisches Museum Wien, Vienna, Austria; 2 Department of Ichthyology and Hydrobiology, Faculty for Biology and Soil, Saint Petersburg State University, St. Petersburg, Russia; 3 Department of Limnology and Bio-Oceanography, University of Vienna, Vienna, Austria; 4 Department of Theoretical Biology, University of Vienna, Vienna, Austria

**Keywords:** nomenclatural acts, availability, lectotype, neotype, *Phoxinus csikii*, *Phoxinus marsilii*

## Abstract

This corrigendum, in the sense of the Glossary of the International Code of Zoological Nomenclature (ICZN 1999, p. 102), is to ensure that the ICZN criteria for the availability of the two new nomenclatural acts, namely the designations of the neotype of *Phoxinus csikii* Hankó, 1922 and the lectotype of *Phoxinus marsilii* Heckel, 1836, are satisfied.

## Introduction

Our original publication (Palandačić et al. 2017) published online on 9 August 2017 (https://bmcevolbiol.biomedcentral.com/articles/10.1186/s12862-017-1032-x) in an electronic-only journal was not registered in ZooBank prior to publication and as such does not contain evidence in the work itself that such registration had occurred. This made the nomenclatural acts in the article (designation of lectotype and neotype) unavailable under the International Code of Zoological Nomenclature (ICZN 1999) according to Art. 8.5.3. of the Amendment to International Code of Zoological Nomenclature (ICZN 2012).

To make the designations of the neotype of *Phoxinus csikii* Hankó, 1922 and the lectotype of *Phoxinus marsilii* Heckel, 1836 available, the present publication contains the Taxonomic Implications section from Palandačić et al. (2017), with some changes to correspond to the Biodiversity Data Journal guidelines and style of a separate publication, and illustrations of the specimens from Palandačić et al. (2017: Additional File 3, Figs S4, S5; original publisher: BioMed Central).

## Designation of a lectotype of *Phoxinus marsilii*

According to ICZN (Art. 74.1, 74.7) a lectotype is herein designated to become the unique bearer of the name of *P. marsilii*. It is properly labelled in the Natural History Museum in Vienna, Austria (NMW) and can be identified by its morphological features described below.

In 1836, two specimens were taken into the collection of the Hof-Naturalien-Cabinett, the forerunner of the NMW, as *Phoxinus marsilii* (Acqu. Nr. 1836.I.20). However, the NMW 51225 sample with this acquisition number contains six specimens. The number and sizes of the specimens Heckel (1836) used to base his description of *P. marsilii* upon is unclear, though it is obvious from the original description that more than one was used. We consider all six specimens as syntypes and designate specimen NMW 51225:2 (Fig. 1) as the lectotype of *P. marsilii* Heckel, 1836. The five paralectotypes are now under NMW 98672.

The type locality of *P. marsilii* (Heckel 1836) was described as clear brooks of the environs of Vienna and beyond (“… in allen klaren Bächen der Wien-Gegend und weiter …”). The lectotype is characterised by the lateral line extending close to the caudal fin base (87 scales in the lateral series: 74 pored and 13 non-pored); two patches of breast scales, not separated by a scaleless area (three rows of scales, 4–6th, confluent); no scales between pelvic and pectoral fins; 8 branched rays in both dorsal and anal fins (last two rays originating on a single pterygiophore); 16/16 branched pectoral fin rays; 7/7 branched pelvic fin rays; total vertebrae, 40 (22 abdominal, and 18 caudal); depth of caudal peduncle, 9.8% standard length (SL), 35% caudal peduncle length and 60.7 % body depth; body depth, 16.1% SL.

The Senckenberg Museum in Frankfurt am Main, Germany (SMF) holds two specimens as syntypes of *P. marsilii* (SMF 1980), received in 1844 from the NMW. From the NMW acquisition sheet for that year it is evident that two specimens labelled *Phoxinus marsilii* Heck. were sent to Prof. Joh. Müller but had been sampled in northern Italy, in brooks at Treviso (Acquisition Nr. 1844.III.3), not in the surroundings of Vienna. As such, they are not syntypes of *P. marsilii*.

## Designation of a neotype of *Phoxinus csikii* and its type locality

*Phoxinus csikii* was described from a karstic brook near Korita (43°00′25″N, 19°58′03″E), Bijelo Polje region in northern Montenegro (*Hankó 1922*). The brook was a sinking stream at the border of the Lim (Drina–Sava–Danube) and Ibar (Zapadna Morava–Danube) river systems. The two syntypes, one juvenile (46 mm total length, TL) and one adult female (75 mm TL), of *P. csikii* were deposited at the Hungarian Natural History Museum in Budapest (MNSB). The original type series is lost (see below). Because several *Phoxinus* species occur in the Danube region (Palandačić et al. 2017), there is an explicit need for the designation of a neotype (Art. 75.3. of ICZN).

We designate the specimen NMW 51266:2, 89.5 mm SL (Fig. 2), now NMW 98673, as the neotype of *Phoxinus csikii*. All qualifying conditions (Art. 75.3 of ICZN) are met: the neotype is designated to clarify the taxonomic status of the species (Art. 75.3.1), and the original description provides a sufficiently full differentiating description of a larger syntype (Art. 75.3.2). The two syntypes of *P. csikii* were donated to MNSB in July 1917 by Ernst [Ernő] Csiki, a Hungarian entomologist and director of the museum at that time. Dr Judit Vörös, the curator of the fish collection in this museum informed that, at present, these specimens are absent from the collection as having been destroyed, probably by a fire in 1956 (Art. 75.3.4.).

The neotype was collected close to the original type locality (Art. 75.3.6.) at Rožaje [Rozaj], Montenegro (42°50′39″N, 20°10′00″E), in the Ibar River, tributary to the Zapadna Morava river, a tributary of the Danube. The neotype is consistent with the original description (Art. 75.3.5) and can be unambiguously recognized (Art. 75.3.3.) through having the following characters: an incomplete lateral line almost continuous to the origin of the anal fin with few single pored scales on the caudal peduncle (last pored scale in the middle of the caudal peduncle); 90 scales in the lateral series (51 pored, 39 non-pored); two patches of breast scales separated distinctly by a scaleless area; the posterior one-third of the area between the pectoral and pelvic origins scaled; eight branched rays in both the dorsal fin and the anal fin (the last two rays originating on a single pterygiophore); 16/15 branched pectoral fin rays; 7/7 branched pelvic fin rays; total vertebrae, 41 (22 abdominal and 19 caudal); depth of the caudal peduncle, 10.3% SL, 40.3% caudal peduncle length and 43% body depth; body depth, 24.8% SL.

## Figures and Tables

**Figure 1.**
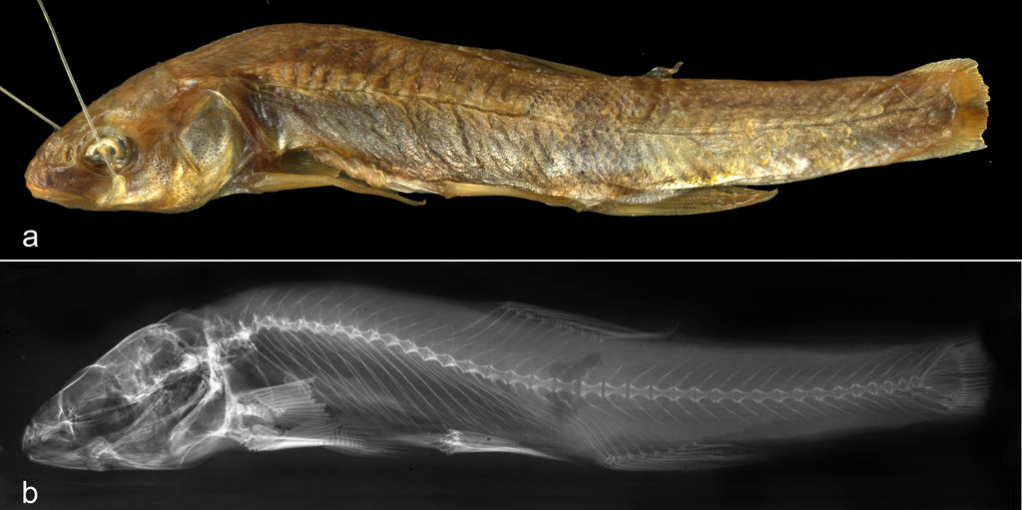
Photo (a) and x-ray (b) of the lectotype *Phoxinus marsilii* (NMW 51225).

**Figure 2.**
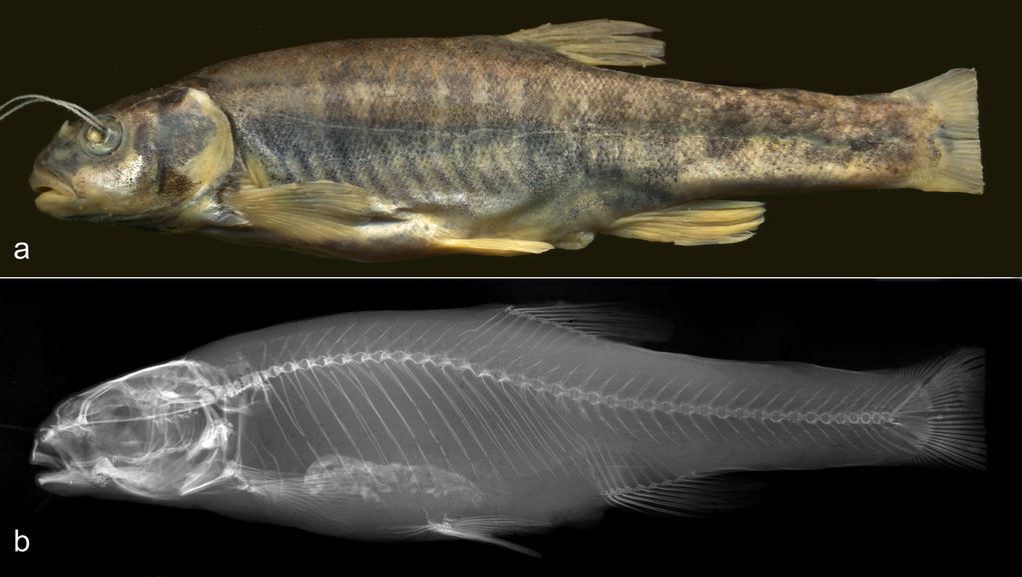
Photo (a) and x-ray (b) of the neotype *Phoxinus csikii* (NMW 98673).
