# Supplementary material for: Contrasting morphology with molecular data: an approach to revision of species complexes based on the example of European Phoxinus (Cyprinidae)
Source: BMC Evol Biol. 2017 Aug 9;17:184. doi: 10.1186/s12862-017-1032-x (PMC5549366; doi:10.1186/s12862-017-1032-x)
Supplement: Supplementary file 3 — ᅟ(DOCX 2760 kb) [file 12862_2017_1032_MOESM3_ESM.docx]

**SUPPLEMENTARY MATERIAL**

**2. Materials and Methods**

**2.1 Samples and dataset**

Fresh samples newly used in this study include samples from Austria (Natural History Museum Vienna – NMW); from Poland donated by Department of Ichthyobiology and Fisheries, University of Agriculture Krakow; from Hungary donated by Department of Aquaculture, Szent Istvan University; from Switzerland, Department for Herpetology and Ichthyology, Natural History Museum of Geneva; from Germany, The Bavarian Natural History Collections; and from Italy, Carmagnola Natural History Museum.

DNA was extracted from fin tissue using DNeasy Blood & Tissue Kit (Qiagen) following the manufacturer’s protocol.

**2.1.1 Mitochondrial DNA**

Polymerase chain reaction (PCR) was performed with primers GluF (5’- AACCACCGTTGTATTCAACTACAA - 3’) and ThrR (5’- ACCTCCGATCTTCGGATTACAAGACCG - 3’; [1]) for cytochrome *b* (cytb), and FishF1 (5'- TCAACCAACCACAAAGACATTGGCAC- 3') and FishR1 (5'- TAGACTTCTGGGTGGCCAAAGAATCA- 3'; [2]) for the barcoding region of cytochrome oxidase I (COI). Reaction volume was 25 μl, with 2.5 μl buffer, 500 μM dNTPs, 1.5 μl (1.5 μM) Mg^2+^, 0.25 μl of each primer (25 pmol μl^-1^), 0.15 μl TopTaq-polymerase (0.5 units) and 2 μl DNA (with approx. concentration of 10 ng μl^-1^). Cycling conditions for cytb were:

initial denaturation 94 ̊C 3 min

| 94 ̊C 30 s |  |
| --- | --- |
| 51 ̊C 30 s | 35 × |
| 72 ̊C 60 s |  |

final extension 72 ̊C 10 min

For COI, annealing was set to 55 C and extension only lasted 45 s. Purification and sequencing (in both directions) of PCR products was performed by LGC Genomics (Berlin, Germany) and Microsynth (Vienna, Austria) with primers used for PCR. The sequences were edited by eye and aligned with MEGA 5.0 [3].

DNA extracted in previous study [4] was used to amplify COI according to protocol described above. Extended table of all used sequences with accompanying information is available in the supplementary Table S2, provided as a separate appendix (Excel spreadsheet).

Generally, sequences from Genbank (Table S2) were of poor quality, exhibiting numerous ambiguous positions. Where multiple sequences from the same locality were available (e.g., Wahlscheid, Germany, Table S2), only those without missing data were used for further analysis.

**2.1.2 Nuclear DNA**

Polymerase chain reaction (PCR) was performed with primers RH28F (5’- TACGTGCCTATGTCCAAYGC - 3’) and RH1039R (5’- TGCTTGTTCATGCAGATGTAGA - 3’; [5] for rhodopsin (RH), and RAG1F (5'- AGCTGTAGTCAGTAYCACAARATG - 3') and RAG9R (5'- GTGTAGAGCCAGTGRTGYTT - 3'; [6]) for recombination activating gene 1 (RAG1). PCR reaction volume was 25 μl, with 2.5 μl of buffer, 2 μl MgCl_2_ (2.0 mM), 1 μl of Enhancer, 500 μM dNTPs, 0.25 μl of each primer and 0.2 μl of AmpliTaq Gold® 360 DNA Polymerase (1 unit).

Cycling conditions in a touch-down PCR reaction for RH were:

initial denaturation 95 ̊C 10 min

| 95 ̊C 40 s |  |
| --- | --- |
| 52 ̊C 60 s | 5 × |
| 72 ̊C 90 s |  |
| 95 ̊C 40 s |  |
| 50 ̊C 40 s | 37 × |
| 72 ̊C 90 s |  |

final extension 72 ̊C 7 min.

Cycling conditions in a touch-down PCR reaction for RAG1 were:

initial denaturation 95 ̊C 10 min

| 95 ̊C 45 s |  |
| --- | --- |
| 58 ̊C 75 s – reducing the temperature 0.5 ̊C per cycle | 10 × |
| 72 ̊C 105 s |  |
| 95 ̊C 45 s |  |
| 53 ̊C 75 s | 37 × |
| 72 ̊C 105 s |  |

final extension 72 ̊C 10 min.

**2.2 Museum material**

Laboratory procedures involving museum material were performed in a DNA clean room with sterilised and UV radiated utensils. DNA was extracted either from one cm^2^ fin tissue or from all five branchial arches from the right side of the specimens. The tissue was air dried to eliminate residual ethanol and DNA extracted with QIAamp® DNA Mini and Blood Mini Kit (Qiagen) following the manufacturer’s protocol. All extractions included extraction controls to ensure there was no contamination of the buffers. Because museum DNA is typically fragmented, we developed additional primers to amplify from 150 – 350 bp long fragments of COI and cytb. Primers were arranged across the regions in a way that adjacent fragments overlap for at least 30 bp - an additional control for contamination. For COI, the complete region was put together (652 bp), while for cytb 590 or 473 bp long parts were obtained, depending on the DNA quality. All PCRs included negative and positive controls. Touch-down PCR protocol was used for all fragments together with increased number of cycles (45). Purification of PCR products was performed with Qiagen PCR purification kit, and purified PCR products were sequenced (in both directions) by LGC Genomics (Berlin, Germany) with PCR primers. After amplification, the fragments were aligned with MEGA 5.0 [3] and composed into a single sequence. COI and cytb fasta files with aligned fragments and primers are available as separate fasta files. Composed sequences were then added to the dataset for phylogenetic and species delimitation analyses.

PCR reaction volume for museum samples was 25 μl, with 2.5 μl of buffer, 2 μl MgCl_2_ (2.0 mM), 1 μl of Enhancer, 500 μM dNTPs, 0.25 μl of each primer (25 pmol μl^-1^; for primer sequences see the Table S3 below) and 0.2 μl of AmpliTaq Gold® 360 DNA Polymerase (1 unit). The volume of the DNA varied according to measured concentration. Usually the aim was 10 - 20 ng μl^-1^ of final concentration in 25 μl reaction.

**Table S3: Primers, corresponding fragment lengths, and PCR conditions used for museum material.** Primer – primer name; Sequence of the primer from 5- to 3- end; Paired with – name of the other primer from primer pair, for FishF1, FishR1, GluF see text above; Product - product length in base pairs; Name - name of the fragment in the fasta file**.**

| Primer | Sequence | Paired with | Product | Name |
| --- | --- | --- | --- | --- |
| **Gene: COI** |  |  |  |  |
| COI_mus_R1 | 5'- CGGGGAAAGGCTATGTCAGG- 3' | FishF1 | 263 bp | C1 |
| COI_mus_F3 | 5'- GCCAATTCTTATTGGCGGATTTGG- 3' | COI_mus_R3 | 252 bp | C2 |
| COI_mus_R3 | 5'- ACACCTGCTAGATGAAGTGAGA- 3' | COI_mus_F3 | 252 bp | C2 |
| COI_mus_F4 | 5'- ATGCCGGTGCATCAGTAGA- 3' | FishR1 | 319 bp | C3 |
| COI_mus_F5 | 5‘- CAGGTTGAACCGTATATCCCCC – 3‘ | COI_mus_R5 | 161 bp | C4 |
| COI_mus_R5 | 5‘- GGAGATGGCTGGGGGTTTCAT- 3‘ | COI_mus_F5 | 161 bp | C4 |
| **Gene: cytb short** |  |  |  |  |
| GLU1 R | 5'-TAATTAACGTCCCGGCAGAT- 3' | GluF | 258 bp | G1 |
| GLU2 F | 5'-TCTGGAACTTTGGTTCTCTTCT**r**GG-3' | GLUFkompl2R | 266 bp | G2 |
| GLU2 R | 5'-AA**y**TACGCCGATGTTTCAGGT-3' | GLUF2F | 266 bp | G2 |
| GLU3 F | 5'- TATTTATATGCATATTGCCCGAGG - 3' | GluFkompl3R | 273 bp | G3 |
| GLU3 R | 5'- AGGAAGTGAAATGCGAAGAATCG - 3' | GluF3F | 273 bp | G3 |
| **Gene: cytb long** |  |  |  |  |
| GLU1 R | 5'-TAATTAACGTCCCGGCAGAT- 3' | GluF | 258 bp | G1 |
| BLAST1 F | 5'-GCACTCGTYGAYCTCCCRAC-3' | BLAST1R | 353 bp | B1 |
| BLAST1 R | 5'-GGAAGGACGTAGCCYACAAA-3' | BLAST1F | 353 bp | B1 |
| BLAST2 F | 5'-TGCCCGGGGTCTTTATTA**y**GG-3' | BLAST2R | 348 bp | B2 |
| BLAST2 R | 5'-TC**y**GAGTTTAATCCGGCGGG-3' | BLAST2F | 348 bp | B2 |

**Cycling conditions**

**Primer pairs: FishF1 + COI_mus_R1, COI_mus_F3 + COI_mus_R3, COI_mus_F4 + FishR1, COI_mus_F5 + COI_mus_R5**

initial denaturation 95 ̊C 10 min

| 95 ̊C 30 s |  |
| --- | --- |
| 53 ̊C 45 s | 5 × |
| 72 ̊C 30 s |  |

| 95 ̊C 30 s |  |
| --- | --- |
| 49 ̊C 30 s | 40 × |
| 72 ̊C 30 s |  |

final extension 72 ̊C 7 min.

**Primer pairs: BLAST1 F + BLAST1 R, GLU1 F + GLU1 R**

initial denaturation 95 ̊C 10 min

| 95 ̊C 30 s |  |
| --- | --- |
| 52 ̊C 30 s | 5 × |
| 72 ̊C 30 s |  |

| 95 ̊C 30 s |  |
| --- | --- |
| 49 ̊C 30 s | 40 × |
| 72 ̊C 30 s |  |

final extension 72 ̊C 7 min.

**Primer pairs: BLAST2 F + BLAST2 R, GLU2 F + GLU2 R, GLU3 F + GLU3 R**

initial denaturation 95 ̊C 10 min

| 95 ̊C 30 s |  |
| --- | --- |
| 53 ̊C 30 s | 5 × |
| 72 ̊C 30 s |  |

| 95 ̊C 30 s |  |
| --- | --- |
| 49 ̊C 30 s | 40 × |
| 72 ̊C 30 s |  |

final extension 72 ̊C 7 min.

**2.3 Mitochondrial DNA**

**2.3.1 Phylogenetic analyses**

To revise putative *Phoxinus* species in Europe, primarily the COI dataset was used for phylogenetic analysis. Besides, phylogenetic reconstruction was performed from three additional data sets: cytb, COI+cytb and COI+partial cytb region corresponding to the shorter length (475 bp) of the cytb fragment amplified from the museum samples. For all alignments, the model of nucleotide substitution was selected using hierarchical likelihood ratio tests implemented by jModelTest v.0.1.1 (Table S4; [7]). Because programs for phylogenetic analyses do not support all the models provided by jModelTest, the closest available model was used (Table S4). For COI+cytb and COI+cytb partial partitions were used, with each gene having a corresponding best model of nucleotide evolution.

**Table S4: The best model of nucleotide substitution** calculated with jModelTest v.0.1.1 [7] and the models used for each analysis. Because the programs for phylogenetic inference usually do not support all the models outputted by jModelTest, the closest available model was used for different analyses. bp – base pair; BIC - Bayesian information criterion; AIC - Akaike information criterion; BEAST – the closest available model used for BEAST analysis; GARLI – the closest available model used for GARLI analysis; PHYML – the closest available model used for PHYML analysis.

| Dataset | Length | BIC | AIC | BEAST | GARLI | PHYML |
| --- | --- | --- | --- | --- | --- | --- |
| COI | 651 bp | TPM3+I+G | TPM3+I+G | SRD06 | / | GTR+G+I |
| cytb | 1091 bp | GTR+G+I | GTR+G+I | GTR+G+I | / | GTR+G+I |
| cytb partial | 473 bp | TrN+G | TPM3+I+G | / | / | GTR+G+I |
| COI+cytb | 1742 bp | / | / | COI: SRD06  cytb: GTR+G+I | COI: TPM3+I+G  cytb: GTR+G+I | / |
| COI+cytb partial | 1124 bp | / | / | COI: SRD06  cytb: HKY+G | COI: TPM3+I+G  cytb: TrN+G | / |

For phylogenetic reconstruction with Bayesian inference (BI) using BEAST 1.8.0 [8], appropriate model for phylogenetic reconstruction for each dataset was determined using path sampling (PS) and stepping-stone (SS) model selection criteria (Table S5; [9, 10]). Because PS and SS analyses are extremely computer exhaustive, and the analyses were cut off in the CIPRES server (https://www.phylo.org/portal2/) after one week, only unique haplotypes were used. In addition to testing best Clocks and Trees Models, Sites Model was also tested (even though the sites model corresponds to some extent to jModelTest analysis). For COI, SRD06 outperformed GTR+G+I for the same Clocks and Trees Model, thus this model was chosen for further analysis (Table S4). After choosing the best model, phylogenetic analysis of COI and cytb was performed with three independent runs (50,000,000 steps) and combined with LogCombiner (part of the BEAST package) once the first 10 % of steps of each run were discharged as a burn-in phase. For COI+cytb and COI+cytb partial, both partitions were loaded to BEAST. Because Clocks and Trees Models are the same, only the Sites Model was unlinked between partitions, and for each gene, the best model was used (SDR06 for COI and GTR+I+G for cytb).

Convergence of each analysis was checked with Tracer 1.6 [11].

**Table S5: Best models for phylogenetic reconstruction** with BEAST as chosen according to the path sampling (PS) and stepping-stone (SS) model selection criteria [9, 10].

| Dataset | Sites Model | Clocks Model | Trees Model |
| --- | --- | --- | --- |
| COI | SDR06, with gamma prior distribution for CP.mu | Strict | Speciation: Birth-Death Process |
| cytb | GTR+G+I | Strict | Speciation: Birth-Death Process |

Phylogenetic trees for COI and cytb were also constructed using Maximum-Likelihood method implemented in PhyML ([12]; http://www.atgc-montpellier.fr/phyml/) with appropriate model of nucleotide substitution (Table S4), SPR & NNI tree improvement, 4 random starting trees (as recommended in the manual) and 500 bootstraps. Because PhyML does not support partitioning, GARLI v.2.01 [13, 14] was used for calculating ML trees of combined dataset COI+cytb and COI+cytb partial. The tree search was performed using 3 search replicates and appropriate evolutionary model; other settings were left default, as specified in the manual. The tree with the best log likelihood was used as the best tree, onto which bootstrap values (after repeating the analysis with 500 bootstrap replicates) were extrapolated. Bootstrap values were summed by the program SumTrees 4.0.0 [15, 16] included in DendroPy 4.0.0.dev package.

Genetic distances between clades detected in the phylogenetic analysis were calculated using MEGA 5.0 [3] as explained below under ABGD chapter.

**2.3.2 Species delimitation**

Species delimitation was performed on COI dataset using three different methods: Automatic Barcode Gap Discovery (ABGD; [17]), General Mixed Yule Coalescent model (GMYC; [18]) and Poisson Tree Processes (PTP; [19]), each of which uses different approach to delimit species. ABGD automatically detects a gap in the distribution of pairwise genetic distances and it was designed to form species hypotheses from huge amounts of data newly available from barcoding projects. Two additional phylogenetic-tree based methods were used, GMYC model for ultrametric trees and PTP model for un-time-calibrated phylogenetic trees. All three methods are widely used for mtDNA. ABGD method was performed on the server (<http://wwwabi.snv.jussieu.fr/public/abgd/abgdweb.html>). Because the sever supports only the simplest nucleotide substitution models, we calculated genetic distance matrices using MEGA 5.0 [3] and the best (closest) model of nucleotide substitution (TPM + I + G). The matrices were uploaded and the delimitation calculated with the default parameters. GMYC and PTP were run with trees constructed with unique haplotypes only, using the webservers <http://species.h-its.org/gmyc/> and <http://species.h-its.org/ptp/>, respectively. According to simulations, the “single threshold” method outperforms “multiple thresholds” method [18], thus only “single threshold” method was performed. The phylogenetic input tree for GMYC was constructed using BEAST 1.8.0 [8]. In their study, Monaghan *et al.* [20] showed that the best models for the input tree to be used with GMYC are relaxed lognormal clock and a coalescent prior, thus we used these parameters to construct our input tree. The analysis was run for 50,000,000 generations, with a tree sampled each 5000 generations. After the convergence of the analysis was checked with Tracer 1.6 [11], a consensus tree was calculated and used as an input tree. The input tree for PTP was constructed using GARLI 2.01 [13, 14] and 1,000 bootstrap replicates.

**Results**

**Table S6: Museum material used in the study.**

Lab ID - Laboratory identification number; NMW – collection inventory number, following the specimen’s number (e.g., _1), last two samples are from the collection of the Museum of Natural History Berlin, Germany, therefore they have the collection number ZBM; Name – the name under which the specimens are kept in the museum, *Phoxinus laevis* is a junior synonym of *P. phoxinus*; Countries: MNE – Montenegro, AT – Austria, UA – Ukraine, D - Germany; Year – the year of collection; Status – type or non-type; Gene - COI – complete region of COI (652 bp), only C1 of COI – only the first fragment was successfully amplified, the complete COI is composed from four fragments (C1 - C4), 590 cytb - 590 bp long fragment of cytb, 473 cytb – 473 bp long fragment of cytb; * - used in previous study [21], see Table S2 for GenBank number.

| Lab ID | NMW | Name | Locality, River | Country | Year | Status | Gene |
| --- | --- | --- | --- | --- | --- | --- | --- |
| 47M | 51266_1 | *Phoxinus laevis* | Rozaje, Ibar | MNE | 1917 |  | COI + 473 cytb |
| 48M | 51266_2 | *P. laevis* | Rozaje, Ibar | MNE | 1917 |  | COI + 473 cytb |
| 66M | 51225_2 | *P. marsilii* | Vienna, ? | AT | 1836 | TYPE | COI + 473 cytb |
| 74M* | 51209_1 | *P. laevis* | Lake Lunz | AT | 1899 |  | COI + 590 bp |
| 75M* | 51216_1 | *P. laevis* | Lake Lunz | AT | 1899 |  | COI + 349 bp |
| 88M | 51266_5 | *P. laevis* | Rozaje, Ibar | MNE | 1917 |  | COI |
| 89M | 51266_6 | *P. laevis* | Rozaje, Ibar | MNE | 1917 |  | COI |
| 93M | 51266_3 | *P. laevis* | Rozaje, Ibar | MNE | 1917 |  | COI |
| 94M | 51266_4 | *P. laevis* | Rozaje, Ibar | MNE | 1917 |  | COI |
| 100M* | 51238_1 | *P. laevis* | Vorokhta, Pruth | UA | 1900 |  | COI + 590 bp |
| 101M* | 51238_2 | *P. laevis* | Vorokhta, Pruth | UA | 1900 |  | COI + 590 bp |
| 102M* | 51238_3 | *P. laevis* | Vorokhta, Pruth | UA | 1900 |  | COI + 590 bp |
| 114M* | 51286_1 | *P. laevis* | Vorokhta, Pruth | UA | 1900 |  | COI |
| 115M* | 51254_2 | *P. laevis* | Vorokhta, Pruth | UA | 1900 |  | COI |
| 116M* | 51260_3 | *P. laevis* | Vorokhta, Pruth | UA | 1900 |  | COI |
| 117M* | 51265_4 | *P. laevis* | Vorokhta, Pruth | UA | 1900 |  | COI |
| 118M* | 51272_6 | *P. laevis* | Vorokhta, Pruth | UA | 1900 |  | COI |
| 173 | ZBM 31261_1 | *P. phoxinus* | Upahl, Stepenitz | D | 1981 |  | only C1 and C2 of COI |
| 174 | ZBM 31261_2 | *P. phoxinus* | Upahl, Stepenitz | D | 1981 |  | only C1 and C2 of COI |

**Phylogenetic analyses**


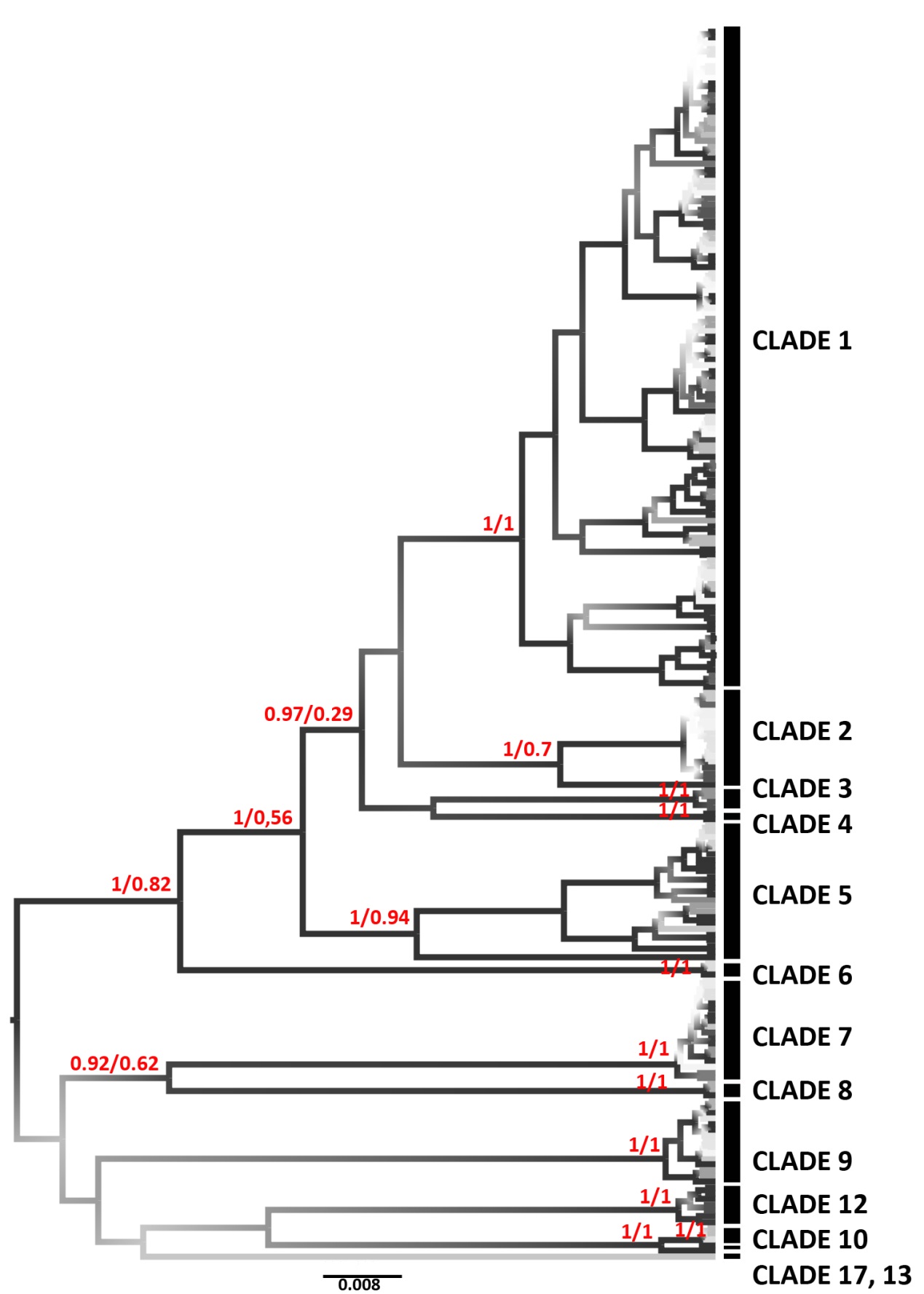


**Figure S1: Phylogenetic reconstruction using cytb dataset.** Phylogenetic tree was constructed from cytochrome *b* collapsed alignment and includes 214 unique haplotypes. The tree was created using Bayesian inference (BI) with BEAST 1.8.0 [8]. Branches carry posterior probabilities (PP) and bootstraps (BS) from the three constructed with the Maximum-Likelihood method (PhyML; [12]). Weakly supported nodes are grey and only PP over 0.9 are shown for the main clades (no sub-clades). -, denotes lack of bootstraps originating from the difference between the BEAST and ML trees.

**
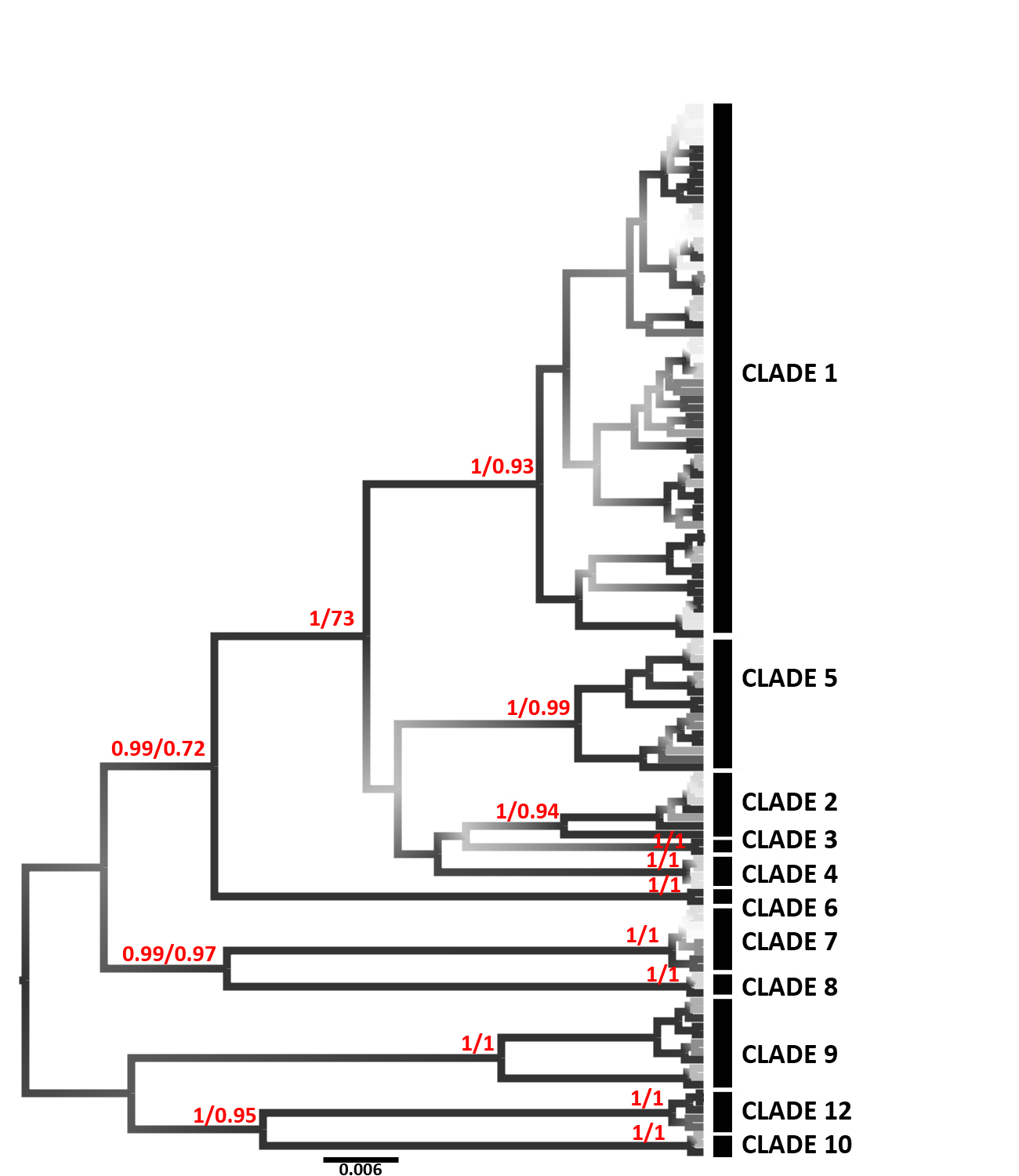
**

**Figure S2: Phylogenetic reconstruction using COI+cytb partial dataset.** Phylogenetic tree was constructed with two partitions, cytochrome oxidase I and cytochrome *b* partial, which denotes the shorter length of the cytb fragment amplified for the museum samples (475 bp). The tree was constructed from collapsed alignment and includes 126 unique haplotypes. The tree was created using Bayesian inference (BI) with BEAST 1.8.0 [8]. Branches carry posterior probabilities (PP) and bootstraps (BS) from the three constructed with the Maximum-Likelihood method (GARLI v.2.01, [13, 14]). Weakly supported nodes are grey and only PP over 0.9 are shown for the main clades (no sub-clades). -, denotes lack of bootstraps originating from the difference between the BEAST and ML trees.

**Table S7: Within group mean genetic distance based on COI barcoding fragment.** The number of base substitutions per site from averaging over all sequence pairs within each group are shown. Analyses were conducted using the Tamura 3-parameter + gamma distribution model. The differences in the composition bias among sequences were considered in evolutionary comparisons [22]. The analysis involved 532 nucleotide sequences with a total of 651 positions and codon positions 1st+2nd+3rd+Noncoding. All positions with less than 95% site coverage were eliminated. Evolutionary analyses were conducted in MEGA5 [3]. The presence of n/c in the results denotes cases in which it was not possible to estimate evolutionary distances (the clade is represented by only one haplotype). Distance – within group mean distance; st.dv - standard error estimate(s).

| (sub)clade | distance | st.dv. |
| --- | --- | --- |
| 1a | 0.0024 | 0.0009 |
| 1b | 0.0029 | 0.0014 |
| 1c | 0.0016 | 0.0009 |
| 1d | 0.0016 | 0.0010 |
| 1e | 0.0009 | 0.0009 |
| 1f | 0.0012 | 0.0008 |
| 2 | 0.0016 | 0.0007 |
| 2a | n/c | n/c |
| 5a | 0.0016 | 0.0004 |
| 5b | 0.0050 | 0.0017 |
| 3 | 0.0006 | 0.0006 |
| 4 | 0 | 0 |
| 6 | 0 | 0 |
| 7 | 0.0019 | 0.0009 |
| 8 | 0.0021 | 0.0014 |
| 9a | 0.0015 | 0.0007 |
| 9b | 0.0008 | 0.0008 |
| 9c | n/c | n/c |
| 10 | 0.0005 | 0.0002 |
| 11 | 0.0018 | 0.0009 |
| 12 | 0.0048 | 0.0019 |
| 13 | 0.0054 | 0.0024 |
| 14 | 0.0042 | 0.0022 |
| 15 | 0 | 0 |
| 17 | n/c | n/c |
| 16 | 0.0041 | 0.0014 |
| 18 | 0.0167 | 0.0057 |

**Table S8: Genetic distances between the subclades based on COI.** The number of base substitutions per site from estimation of net average between groups of sequences are shown. Standard error estimate(s) are shown above the diagonal. Analyses were conducted using the Tamura 3-parameter + gamma distribution model. The differences in the composition bias among sequences were considered in evolutionary comparisons [22]. The analysis involved 532 nucleotide sequences with a total of 651 positions and codon positions 1st+2nd+3rd+Noncoding. All positions with less than 95% site coverage were eliminated. Evolutionary analyses were conducted in MEGA5 [3].

| **clade** | **1a** | **1b** | **1c** | **1d** | **1e** | **1f** | **2** | **2a** | **3** | **4** | **5a** | **5b** | **6** | **7** | **8** | **9a** | **9b** | **9c** | **10** | **11** | **12** | **13** | **14** | **15** | **16** | **17** | **18** |
| --- | --- | --- | --- | --- | --- | --- | --- | --- | --- | --- | --- | --- | --- | --- | --- | --- | --- | --- | --- | --- | --- | --- | --- | --- | --- | --- | --- |
| **1a** |  | 0 | 0 | 0.01 | 0 | 0 | 0.01 | 0.01 | 0.01 | 0.01 | 0.01 | 0.01 | 0.01 | 0.01 | 0.01 | 0.01 | 0.02 | 0.02 | 0.02 | 0.01 | 0.02 | 0.01 | 0.01 | 0.01 | 0.01 | 0.02 | 0.02 |
| **1b** | 0.01 |  | 0 | 0.01 | 0 | 0 | 0.01 | 0.01 | 0.01 | 0.01 | 0.01 | 0.01 | 0.01 | 0.01 | 0.01 | 0.01 | 0.01 | 0.01 | 0.01 | 0.01 | 0.01 | 0.01 | 0.01 | 0.01 | 0.01 | 0.02 | 0.02 |
| **1c** | 0.01 | 0 |  | 0.01 | 0 | 0 | 0.01 | 0.01 | 0.01 | 0.01 | 0.01 | 0.01 | 0.01 | 0.01 | 0.01 | 0.01 | 0.02 | 0.02 | 0.02 | 0.01 | 0.02 | 0.01 | 0.01 | 0.01 | 0.01 | 0.02 | 0.02 |
| **1d** | 0.02 | 0.02 | 0.02 |  | 0 | 0 | 0.01 | 0.01 | 0.01 | 0.01 | 0.01 | 0.01 | 0.01 | 0.01 | 0.01 | 0.01 | 0.02 | 0.02 | 0.02 | 0.01 | 0.02 | 0.01 | 0.01 | 0.01 | 0.01 | 0.02 | 0.02 |
| **1e** | 0.01 | 0.01 | 0.01 | 0.01 |  | 0 | 0.01 | 0.01 | 0.01 | 0.01 | 0.01 | 0.01 | 0.01 | 0.01 | 0.01 | 0.01 | 0.01 | 0.01 | 0.01 | 0.01 | 0.01 | 0.01 | 0.01 | 0.00 | 0.01 | 0.01 | 0.01 |
| **1f** | 0.01 | 0.01 | 0.01 | 0.01 | 0 |  | 0.01 | 0.01 | 0.01 | 0.01 | 0.01 | 0.01 | 0.01 | 0.01 | 0.01 | 0.01 | 0.01 | 0.01 | 0.01 | 0.01 | 0.01 | 0.01 | 0.01 | 0.01 | 0.01 | 0.02 | 0.01 |
| **2** | 0.03 | 0.03 | 0.03 | 0.03 | 0.02 | 0.03 |  | 0.01 | 0.01 | 0.01 | 0.01 | 0.01 | 0.01 | 0.01 | 0.01 | 0.01 | 0.01 | 0.01 | 0.01 | 0.01 | 0.02 | 0.01 | 0.01 | 0.01 | 0.01 | 0.02 | 0.02 |
| **2a** | 0.04 | 0.03 | 0.04 | 0.04 | 0.03 | 0.03 | 0.02 |  | 0.01 | 0.01 | 0.01 | 0.01 | 0.01 | 0.01 | 0.01 | 0.01 | 0.01 | 0.01 | 0.01 | 0.01 | 0.01 | 0.02 | 0.01 | 0.01 | 0.01 | 0.02 | 0.02 |
| **3** | 0.03 | 0.02 | 0.03 | 0.02 | 0.02 | 0.02 | 0.02 | 0.02 |  | 0.01 | 0.01 | 0.01 | 0.01 | 0.01 | 0.01 | 0.01 | 0.01 | 0.01 | 0.01 | 0.01 | 0.01 | 0.01 | 0.01 | 0.01 | 0.01 | 0.02 | 0.01 |
| **4** | 0.03 | 0.02 | 0.03 | 0.03 | 0.02 | 0.02 | 0.02 | 0.03 | 0.02 |  | 0.01 | 0.01 | 0.01 | 0.01 | 0.01 | 0.01 | 0.01 | 0.01 | 0.01 | 0.01 | 0.01 | 0.01 | 0.01 | 0.01 | 0.01 | 0.01 | 0.02 |
| **5a** | 0.02 | 0.02 | 0.02 | 0.02 | 0.02 | 0.02 | 0.02 | 0.03 | 0.02 | 0.02 |  | 0 | 0.01 | 0.01 | 0.01 | 0.01 | 0.01 | 0.01 | 0.01 | 0.01 | 0.02 | 0.01 | 0.01 | 0.01 | 0.01 | 0.02 | 0.02 |
| **5b** | 0.03 | 0.02 | 0.02 | 0.02 | 0.02 | 0.02 | 0.03 | 0.03 | 0.02 | 0.02 | 0.00 |  | 0.01 | 0.01 | 0.01 | 0.01 | 0.01 | 0.01 | 0.01 | 0.01 | 0.01 | 0.01 | 0.01 | 0.01 | 0.01 | 0.02 | 0.02 |
| **6** | 0.04 | 0.04 | 0.04 | 0.04 | 0.03 | 0.03 | 0.04 | 0.05 | 0.03 | 0.04 | 0.02 | 0.03 |  | 0.01 | 0.01 | 0.01 | 0.01 | 0.01 | 0.01 | 0.01 | 0.02 | 0.01 | 0.01 | 0.01 | 0.01 | 0.02 | 0.02 |
| **7** | 0.06 | 0.06 | 0.06 | 0.06 | 0.05 | 0.05 | 0.06 | 0.06 | 0.05 | 0.06 | 0.06 | 0.05 | 0.06 |  | 0.01 | 0.01 | 0.02 | 0.01 | 0.02 | 0.01 | 0.01 | 0.01 | 0.01 | 0.01 | 0.01 | 0.02 | 0.02 |
| **8** | 0.05 | 0.05 | 0.05 | 0.05 | 0.04 | 0.04 | 0.05 | 0.05 | 0.04 | 0.04 | 0.05 | 0.05 | 0.05 | 0.04 |  | 0.01 | 0.01 | 0.01 | 0.01 | 0.01 | 0.01 | 0.01 | 0.01 | 0.01 | 0.01 | 0.02 | 0.02 |
| **9a** | 0.06 | 0.05 | 0.06 | 0.06 | 0.04 | 0.05 | 0.05 | 0.06 | 0.05 | 0.05 | 0.05 | 0.05 | 0.05 | 0.06 | 0.05 |  | 0.01 | 0.00 | 0.01 | 0.01 | 0.01 | 0.01 | 0.01 | 0.01 | 0.01 | 0.02 | 0.01 |
| **9b** | 0.07 | 0.06 | 0.07 | 0.07 | 0.05 | 0.06 | 0.06 | 0.07 | 0.05 | 0.06 | 0.06 | 0.05 | 0.06 | 0.07 | 0.06 | 0.02 |  | 0.01 | 0.01 | 0.01 | 0.01 | 0.01 | 0.01 | 0.01 | 0.01 | 0.01 | 0.01 |
| **9c** | 0.07 | 0.06 | 0.07 | 0.07 | 0.05 | 0.06 | 0.06 | 0.07 | 0.05 | 0.06 | 0.06 | 0.06 | 0.06 | 0.07 | 0.05 | 0.01 | 0.02 |  | 0.01 | 0.01 | 0.01 | 0.01 | 0.01 | 0.01 | 0.01 | 0.02 | 0.01 |
| **10** | 0.08 | 0.07 | 0.07 | 0.07 | 0.06 | 0.06 | 0.07 | 0.06 | 0.06 | 0.07 | 0.07 | 0.06 | 0.06 | 0.08 | 0.06 | 0.05 | 0.05 | 0.05 |  | 0.01 | 0.01 | 0.01 | 0.01 | 0.01 | 0.01 | 0.02 | 0.01 |
| **11** | 0.06 | 0.05 | 0.06 | 0.06 | 0.04 | 0.05 | 0.06 | 0.06 | 0.05 | 0.05 | 0.06 | 0.06 | 0.05 | 0.07 | 0.05 | 0.04 | 0.05 | 0.04 | 0.04 |  | 0.01 | 0.01 | 0.01 | 0.01 | 0.01 | 0.01 | 0.01 |
| **12** | 0.08 | 0.07 | 0.08 | 0.07 | 0.06 | 0.06 | 0.07 | 0.07 | 0.06 | 0.06 | 0.07 | 0.07 | 0.08 | 0.07 | 0.06 | 0.06 | 0.06 | 0.06 | 0.04 | 0.06 |  | 0.02 | 0.02 | 0.01 | 0.01 | 0.01 | 0.02 |
| **13** | 0.06 | 0.06 | 0.06 | 0.06 | 0.05 | 0.06 | 0.06 | 0.07 | 0.06 | 0.06 | 0.06 | 0.05 | 0.06 | 0.06 | 0.06 | 0.06 | 0.06 | 0.07 | 0.05 | 0.06 | 0.08 |  | 0.02 | 0.01 | 0.01 | 0.02 | 0.02 |
| **14** | 0.03 | 0.02 | 0.03 | 0.03 | 0.02 | 0.02 | 0.03 | 0.03 | 0.02 | 0.02 | 0.02 | 0.02 | 0.04 | 0.06 | 0.05 | 0.05 | 0.06 | 0.06 | 0.07 | 0.06 | 0.07 | 0.07 |  | 0.01 | 0.01 | 0.02 | 0.02 |
| **15** | 0.02 | 0.02 | 0.02 | 0.02 | 0.01 | 0.01 | 0.02 | 0.03 | 0.02 | 0.02 | 0.02 | 0.02 | 0.03 | 0.05 | 0.04 | 0.05 | 0.06 | 0.06 | 0.06 | 0.05 | 0.07 | 0.06 | 0.02 |  | 0.01 | 0.02 | 0.01 |
| **16** | 0.06 | 0.05 | 0.06 | 0.05 | 0.04 | 0.05 | 0.05 | 0.05 | 0.05 | 0.04 | 0.05 | 0.05 | 0.05 | 0.05 | 0.05 | 0.05 | 0.05 | 0.05 | 0.05 | 0.05 | 0.05 | 0.06 | 0.06 | 0.05 |  | 0.01 | 0.02 |
| **17** | 0.09 | 0.08 | 0.09 | 0.08 | 0.07 | 0.07 | 0.08 | 0.07 | 0.07 | 0.07 | 0.08 | 0.08 | 0.08 | 0.09 | 0.08 | 0.07 | 0.07 | 0.08 | 0.08 | 0.06 | 0.06 | 0.09 | 0.08 | 0.07 | 0.07 |  | 0.01 |
| **18** | 0.08 | 0.07 | 0.08 | 0.08 | 0.06 | 0.07 | 0.08 | 0.07 | 0.07 | 0.08 | 0.08 | 0.07 | 0.07 | 0.09 | 0.07 | 0.06 | 0.04 | 0.06 | 0.07 | 0.06 | 0.07 | 0.08 | 0.07 | 0.07 | 0.08 | 0.05 |  |

**Table S9a: Within group mean genetic distance based on COI.** The number of base substitutions per site from averaging over all sequence pairs within each group are shown. Analyses were conducted using the Tamura 3-parameter + gamma distribution model. The differences in the composition bias among sequences were considered in evolutionary comparisons [22]. The analysis involved 532 nucleotide sequences with a total of 651 positions and codon positions 1st+2nd+3rd+Noncoding. All positions with less than 95 % site coverage were eliminated. Evolutionary analyses were conducted in MEGA5 [3]. The presence of n/c in the results denotes cases in which it was not possible to estimate evolutionary distances (the clade is represented by only one haplotype). Distance – within group mean distance; st.dv - standard error estimate(s).

**Table S9b: Genetic distances between the clades based on COI.** The number of base substitutions per site from estimation of net average between groups of sequences are shown. Standard error estimate(s) are shown above the diagonal. For other see Table S8a caption.

**a b**

| **clade** | **distance** | **st.dv.** |  | **clade** | **1** | **2** | **3** | **4** | **5** | **6** | **7** | **8** | **9** | **10** | **11** | **12** | **13** | **14** | **15** | **17** | **16** | **18** |
| --- | --- | --- | --- | --- | --- | --- | --- | --- | --- | --- | --- | --- | --- | --- | --- | --- | --- | --- | --- | --- | --- | --- |
| **1** | 0.009 | 0.002 |  | **1** |  | 0.01 | 0.01 | 0 | 0 | 0.01 | 0.01 | 0.01 | 0.01 | 0.01 | 0.01 | 0.01 | 0.01 | 0 | 0 | 0.01 | 0.01 | 0.01 |
| **2** | 0.003 | 0.001 |  | **2** | 0.02 |  | 0 | 0.01 | 0 | 0.01 | 0.01 | 0.01 | 0.01 | 0.01 | 0.01 | 0.01 | 0.01 | 0.01 | 0.01 | 0.01 | 0.01 | 0.01 |
| **3** | 0.001 | 0.001 |  | **3** | 0.02 | 0.01 |  | 0 | 0 | 0.01 | 0.01 | 0.01 | 0.01 | 0.01 | 0.01 | 0.01 | 0.01 | 0.01 | 0 | 0.01 | 0.01 | 0.01 |
| **4** | 0 | 0 |  | **4** | 0.02 | 0.02 | 0.02 |  | 0 | 0.01 | 0.01 | 0.01 | 0.01 | 0.01 | 0.01 | 0.01 | 0.01 | 0.01 | 0 | 0.01 | 0.01 | 0.01 |
| **5** | 0.004 | 0.001 |  | **5** | 0.02 | 0.02 | 0.02 | 0.02 |  | 0.01 | 0.01 | 0.01 | 0.01 | 0.01 | 0.01 | 0.01 | 0.01 | 0 | 0 | 0.01 | 0.01 | 0.01 |
| **6** | 0 | 0 |  | **6** | 0.03 | 0.04 | 0.03 | 0.03 | 0.02 |  | 0.01 | 0.01 | 0.01 | 0.01 | 0.01 | 0.01 | 0.01 | 0.01 | 0.01 | 0.01 | 0.01 | 0.01 |
| **7** | 0.002 | 0.001 |  | **7** | 0.04 | 0.04 | 0.04 | 0.04 | 0.04 | 0.04 |  | 0.01 | 0.01 | 0.01 | 0.01 | 0.01 | 0.01 | 0.01 | 0.01 | 0.01 | 0.01 | 0.01 |
| **8** | 0.002 | 0.001 |  | **8** | 0.04 | 0.04 | 0.03 | 0.04 | 0.04 | 0.04 | 0.03 |  | 0.01 | 0.01 | 0.01 | 0.01 | 0.01 | 0.01 | 0.01 | 0.01 | 0.01 | 0.01 |
| **9** | 0.008 | 0.002 |  | **9** | 0.04 | 0.04 | 0.04 | 0.04 | 0.04 | 0.04 | 0.04 | 0.04 |  | 0.01 | 0.01 | 0.01 | 0.01 | 0.01 | 0.01 | 0.01 | 0.01 | 0.01 |
| **10** | 0 | 0 |  | **10** | 0.05 | 0.05 | 0.05 | 0.05 | 0.05 | 0.05 | 0.06 | 0.05 | 0.04 |  | 0.01 | 0.01 | 0.01 | 0.01 | 0.01 | 0.01 | 0.01 | 0.01 |
| **11** | 0.002 | 0.001 |  | **11** | 0.04 | 0.04 | 0.04 | 0.04 | 0.04 | 0.04 | 0.05 | 0.04 | 0.03 | 0.04 |  | 0.01 | 0.01 | 0.01 | 0.01 | 0.01 | 0.01 | 0.01 |
| **12** | 0.005 | 0.002 |  | **12** | 0.05 | 0.05 | 0.05 | 0.05 | 0.05 | 0.05 | 0.05 | 0.05 | 0.05 | 0.03 | 0.04 |  | 0.01 | 0.01 | 0.01 | 0.01 | 0.01 | 0.01 |
| **13** | 0.005 | 0.002 |  | **13** | 0.04 | 0.05 | 0.05 | 0.05 | 0.04 | 0.04 | 0.05 | 0.04 | 0.05 | 0.04 | 0.04 | 0.06 |  | 0.01 | 0.01 | 0.01 | 0.01 | 0.01 |
| **14** | 0.004 | 0.002 |  | **14** | 0.02 | 0.02 | 0.02 | 0.02 | 0.02 | 0.03 | 0.04 | 0.04 | 0.04 | 0.05 | 0.04 | 0.05 | 0.05 |  | 0 | 0.01 | 0.01 | 0.01 |
| **15** | 0 | 0 |  | **15** | 0.02 | 0.02 | 0.02 | 0.02 | 0.02 | 0.02 | 0.04 | 0.03 | 0.04 | 0.05 | 0.04 | 0.05 | 0.04 | 0.01 |  | 0.01 | 0.01 | 0.01 |
| **16** | n/c | n/c |  | **16** | 0.04 | 0.04 | 0.04 | 0.04 | 0.04 | 0.04 | 0.04 | 0.04 | 0.04 | 0.04 | 0.04 | 0.04 | 0.04 | 0.04 | 0.04 |  | 0.01 | 0.01 |
| **17** | 0.004 | 0.001 |  | **17** | 0.06 | 0.06 | 0.05 | 0.05 | 0.06 | 0.06 | 0.06 | 0.06 | 0.05 | 0.06 | 0.05 | 0.04 | 0.06 | 0.06 | 0.05 | 0.05 |  | 0.01 |
| **18** | 0.017 | 0.005 |  | **18** | 0.05 | 0.05 | 0.05 | 0.05 | 0.05 | 0.05 | 0.06 | 0.05 | 0.04 | 0.05 | 0.05 | 0.05 | 0.06 | 0.05 | 0.05 | 0.05 | 0.04 |  |

**Table S10a: Within group mean genetic distance based on cytb.** The number of base substitutions per site from averaging over all sequence pairs within each group are shown. Analyses were conducted using the Maximum Composite Likelihood + gamma distribution model. The differences in the composition bias among sequences were considered in evolutionary comparisons [22]. The analysis involved 383 nucleotide sequences with a total of 1091 positions and codon positions 1st+2nd+3rd+Noncoding. All positions with less than 95% site coverage were eliminated. Evolutionary analyses were conducted in MEGA5 [3]. The presence of n/c in the results denotes cases in which it was not possible to estimate evolutionary distances (the clade is represented by only one haplotype). Distance – within group mean distance; st.dv - standard error estimate(s).

**Table S9b: Genetic distances between the clades based on cytb.** The number of base substitutions per site from estimation of net average between groups of sequences are shown. Standard error estimate(s) are shown above the diagonal. For other see Table S9a caption.

**a b**

| **clade** | **distance** | **st.dv.** |  | **clade** | **1** | **2** | **3** | **4** | **5b** | **6** | **7** | **8** | **9a** | **10** | **12** | **13** | **16** |
| --- | --- | --- | --- | --- | --- | --- | --- | --- | --- | --- | --- | --- | --- | --- | --- | --- | --- |
| **1** | 0.029 | 0.003 |  | **1** |  | 0,01 | 0,01 | 0,01 | 0,01 | 0,02 | 0,02 | 0,02 | 0,02 | 0,02 | 0,02 | 0,03 | 0,03 |
| **2** | 0.006 | 0.001 |  | **2** | 0,06 |  | 0,01 | 0,01 | 0,01 | 0,02 | 0,02 | 0,02 | 0,02 | 0,02 | 0,02 | 0,03 | 0,02 |
| **3** | 0.003 | 0.001 |  | **3** | 0,08 | 0,06 |  | 0,01 | 0,01 | 0,02 | 0,02 | 0,02 | 0,02 | 0,02 | 0,02 | 0,03 | 0,03 |
| **4** | 0.002 | 0.001 |  | **4** | 0,08 | 0,05 | 0,06 |  | 0,01 | 0,02 | 0,02 | 0,02 | 0,02 | 0,02 | 0,02 | 0,03 | 0,03 |
| **5** | 0.027 | 0.004 |  | **5** | 0,10 | 0,08 | 0,09 | 0,08 |  | 0,02 | 0,03 | 0,03 | 0,03 | 0,03 | 0,02 | 0,03 | 0,03 |
| **6** | 0.002 | 0.001 |  | **6** | 0,12 | 0,09 | 0,12 | 0,11 | 0,15 |  | 0,03 | 0,03 | 0,03 | 0,03 | 0,03 | 0,03 | 0,03 |
| **7** | 0.004 | 0.001 |  | **7** | 0,15 | 0,11 | 0,14 | 0,14 | 0,16 | 0,14 |  | 0,02 | 0,03 | 0,03 | 0,02 | 0,03 | 0,03 |
| **8** | 0.001 | 0.001 |  | **8** | 0,16 | 0,13 | 0,14 | 0,16 | 0,18 | 0,17 | 0,14 |  | 0,03 | 0,03 | 0,02 | 0,03 | 0,03 |
| **9** | 0.006 | 0.001 |  | **9a** | 0,13 | 0,12 | 0,13 | 0,13 | 0,16 | 0,15 | 0,16 | 0,16 |  | 0,02 | 0,02 | 0,03 | 0,03 |
| **10** | 0.002 | 0.001 |  | **10** | 0,16 | 0,13 | 0,15 | 0,15 | 0,17 | 0,17 | 0,16 | 0,16 | 0,15 |  | 0,02 | 0,02 | 0,00 |
| **12** | 0.006 | 0.002 |  | **12** | 0,12 | 0,12 | 0,13 | 0,13 | 0,12 | 0,18 | 0,14 | 0,14 | 0,12 | 0,12 |  | 0,03 | 0,02 |
| **13** | n/c | n/c |  | **13** | 0,18 | 0,15 | 0,17 | 0,16 | 0,19 | 0,18 | 0,18 | 0,18 | 0,18 | 0,12 | 0,17 |  | 0,02 |
| **16** | n/c | n/c |  | **16** | 0,17 | 0,14 | 0,16 | 0,16 | 0,17 | 0,19 | 0,17 | 0,17 | 0,16 | 0,01 | 0,10 | 0,13 |  |

**Table S11: Genetic distances between the subclades based on cytb.** For other see Table S9 caption.

| **clade** | **1a** | **1b** | **1c** | **1d** | **1e** | **1f** | **2** | **3** | **4** | **5a** | **5b** | **6** | **7** | **8** | **9a** | **10** | **12** | **13** | **16** |
| --- | --- | --- | --- | --- | --- | --- | --- | --- | --- | --- | --- | --- | --- | --- | --- | --- | --- | --- | --- |
| **1a** |  | 0.00 | 0.01 | 0.01 | 0.01 | 0.01 | 0.01 | 0.01 | 0.01 | 0.02 | 0.02 | 0.02 | 0.02 | 0.02 | 0.02 | 0.02 | 0.02 | 0.03 | 0.03 |
| **1b** | 0.03 |  | 0.01 | 0.01 | 0.01 | 0.01 | 0.01 | 0.01 | 0.01 | 0.01 | 0.02 | 0.02 | 0.02 | 0.02 | 0.02 | 0.03 | 0.02 | 0.03 | 0.03 |
| **1c** | 0.03 | 0.03 |  | 0.01 | 0.01 | 0.01 | 0.01 | 0.01 | 0.01 | 0.02 | 0.02 | 0.02 | 0.02 | 0.03 | 0.02 | 0.02 | 0.02 | 0.03 | 0.03 |
| **1d** | 0.04 | 0.04 | 0.04 |  | 0.00 | 0.01 | 0.01 | 0.01 | 0.01 | 0.02 | 0.02 | 0.02 | 0.02 | 0.03 | 0.02 | 0.03 | 0.02 | 0.03 | 0.03 |
| **1e** | 0.04 | 0.04 | 0.04 | 0.03 |  | 0.01 | 0.01 | 0.01 | 0.01 | 0.02 | 0.02 | 0.02 | 0.02 | 0.02 | 0.02 | 0.02 | 0.02 | 0.03 | 0.03 |
| **1f** | 0.04 | 0.04 | 0.04 | 0.03 | 0.03 |  | 0.01 | 0.01 | 0.01 | 0.02 | 0.02 | 0.02 | 0.02 | 0.03 | 0.02 | 0.03 | 0.02 | 0.03 | 0.03 |
| **2** | 0.06 | 0.06 | 0.05 | 0.07 | 0.07 | 0.06 |  | 0.01 | 0.01 | 0.01 | 0.01 | 0.02 | 0.02 | 0.02 | 0.02 | 0.02 | 0.02 | 0.03 | 0.02 |
| **3** | 0.08 | 0.09 | 0.08 | 0.08 | 0.08 | 0.08 | 0.06 |  | 0.01 | 0.01 | 0.01 | 0.02 | 0.02 | 0.02 | 0.02 | 0.02 | 0.02 | 0.03 | 0.03 |
| **4** | 0.07 | 0.08 | 0.07 | 0.08 | 0.09 | 0.08 | 0.05 | 0.06 |  | 0.01 | 0.01 | 0.02 | 0.02 | 0.02 | 0.02 | 0.03 | 0.02 | 0.03 | 0.03 |
| **5a** | 0.09 | 0.09 | 0.09 | 0.10 | 0.10 | 0.09 | 0.08 | 0.08 | 0.08 |  | 0.01 | 0.02 | 0.03 | 0.03 | 0.03 | 0.03 | 0.02 | 0.03 | 0.03 |
| **5b** | 0.10 | 0.10 | 0.10 | 0.10 | 0.10 | 0.10 | 0.08 | 0.09 | 0.09 | 0.04 |  | 0.02 | 0.03 | 0.03 | 0.03 | 0.03 | 0.02 | 0.04 | 0.03 |
| **6** | 0.12 | 0.12 | 0.12 | 0.13 | 0.12 | 0.12 | 0.09 | 0.12 | 0.11 | 0.14 | 0.15 |  | 0.03 | 0.03 | 0.03 | 0.03 | 0.03 | 0.03 | 0.03 |
| **7** | 0.15 | 0.15 | 0.14 | 0.15 | 0.14 | 0.15 | 0.11 | 0.14 | 0.14 | 0.16 | 0.17 | 0.14 |  | 0.02 | 0.03 | 0.03 | 0.02 | 0.03 | 0.03 |
| **8** | 0.15 | 0.16 | 0.16 | 0.18 | 0.16 | 0.18 | 0.13 | 0.14 | 0.16 | 0.18 | 0.18 | 0.17 | 0.14 |  | 0.03 | 0.03 | 0.03 | 0.03 | 0.03 |
| **9a** | 0.14 | 0.13 | 0.12 | 0.14 | 0.14 | 0.14 | 0.12 | 0.13 | 0.13 | 0.16 | 0.16 | 0.15 | 0.16 | 0.16 |  | 0.02 | 0.02 | 0.03 | 0.03 |
| **10** | 0.16 | 0.16 | 0.15 | 0.17 | 0.16 | 0.17 | 0.13 | 0.15 | 0.15 | 0.17 | 0.17 | 0.17 | 0.16 | 0.16 | 0.15 |  | 0.02 | 0.02 | 0.00 |
| **12** | 0.12 | 0.12 | 0.12 | 0.13 | 0.12 | 0.12 | 0.12 | 0.13 | 0.13 | 0.12 | 0.12 | 0.18 | 0.14 | 0.14 | 0.12 | 0.12 |  | 0.03 | 0.02 |
| **13** | 0.18 | 0.17 | 0.17 | 0.20 | 0.19 | 0.18 | 0.15 | 0.17 | 0.16 | 0.19 | 0.20 | 0.18 | 0.18 | 0.18 | 0.18 | 0.12 | 0.17 |  | 0.03 |
| **16** | 0.17 | 0.17 | 0.16 | 0.19 | 0.17 | 0.18 | 0.14 | 0.16 | 0.16 | 0.17 | 0.18 | 0.19 | 0.17 | 0.17 | 0.16 | 0.01 | 0.10 | 0.13 |  |

**Table S12: Species delimitation results.** Species delimitation was performed on COI dataset using three different methods: Automatic Barcode Gap Discovery (ABGD; [17]), General Mixed Yule Coalescent model (GMYC; [18]) and Poisson Tree Processes (PTP; [19]), each of which uses different approach to delimit species. As a basis, results from ABGD were used, and remarks are written where the results do not coincide with other two methods. 🗸 - means it is the same as in ABGD.

| **ABGD** | **GMYC** | **PTP** |
| --- | --- | --- |
| 1a | 🗸 | One group |
| 1b+1c+1e+1f | (1b+1c) (1e+1f) |  |
| 1d | 🗸 | 🗸 |
| 2 | 🗸 | 🗸 |
| 2a | 🗸 | 🗸 |
| 3 | 🗸 | 🗸 |
| 4 | 🗸 | 🗸 |
| 5 | 5a, 5b | 🗸 |
| 6 | 🗸 | 🗸 |
| 7 | 🗸 | 🗸 |
| 8 | 🗸 | 🗸 |
| 9a | 🗸 | 🗸 9a HQ960812 is included |
| 9b | 🗸 | 🗸 |
| 9a HQ960812  Beskydy, Slavic River CZ | 🗸 | Included in 9a |
| 10 | 🗸 | 🗸 |
| 11 | 🗸 | 🗸 |
| 12 | 🗸 | 🗸 |
| 13 | 🗸 | 13a (KU729252 and KU729253) + 13b (KJ554128, KJ554275 and KJ554288) |
| 14 | 🗸 | 🗸 |
| 15 | 🗸 | 🗸 |
| 16 | 🗸 | 🗸 |
| 16 KU729260  Volga, Russia | 🗸 | 🗸 |
| 17 | 🗸 | 🗸 |
| 18 | 🗸 | 🗸 |
| 18 | 🗸 | 🗸 |

**Figure S3**: A haplotype network created from 841 bp long part of recombination activating gene 1 with new samples, first presented herein, and samples from previous study [4]. The network was constructed from 132 sequences, for which the gametic phase was determined using Phase 2.1 [23, 24], implemented in DnaSP 5.10 [25]; resulting in 264 haplotypes. The algorithm used was median-joining [26] implemented in Network 5.1 (www.fluxus-engineering.com) with default settings.


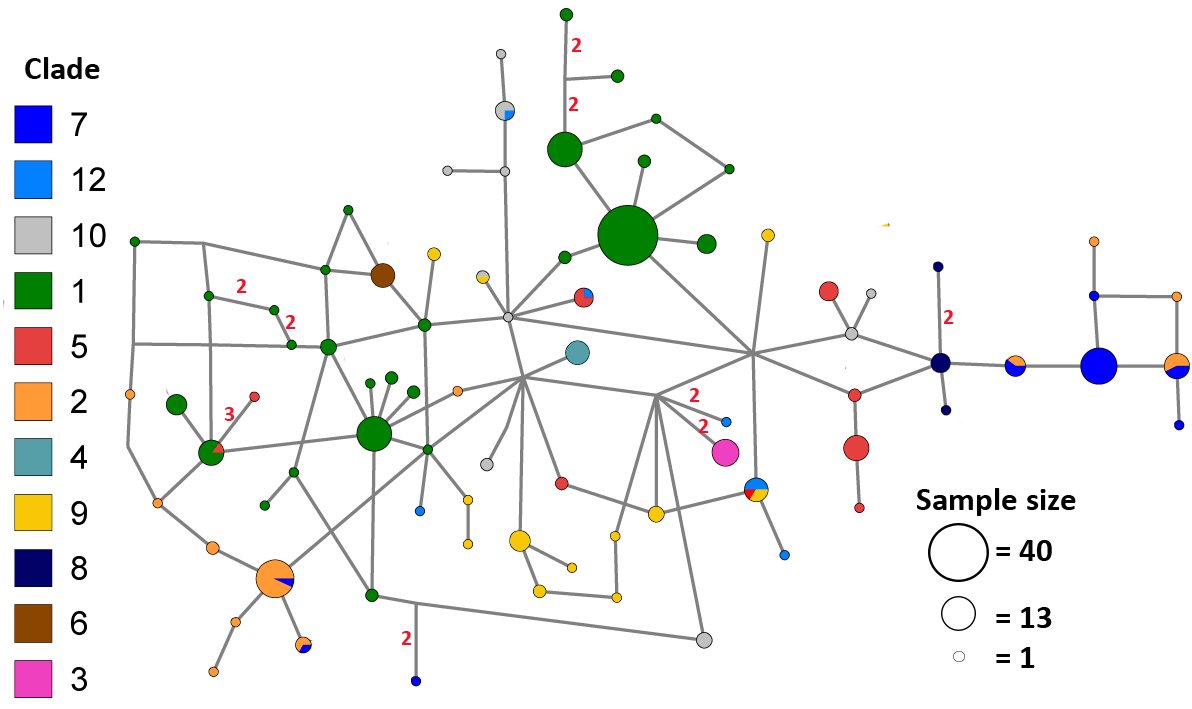


**Figure S4**: Photo (a) and x-ray (b) of the lectotype *Phoxinus marsilii* (NMW-51225).


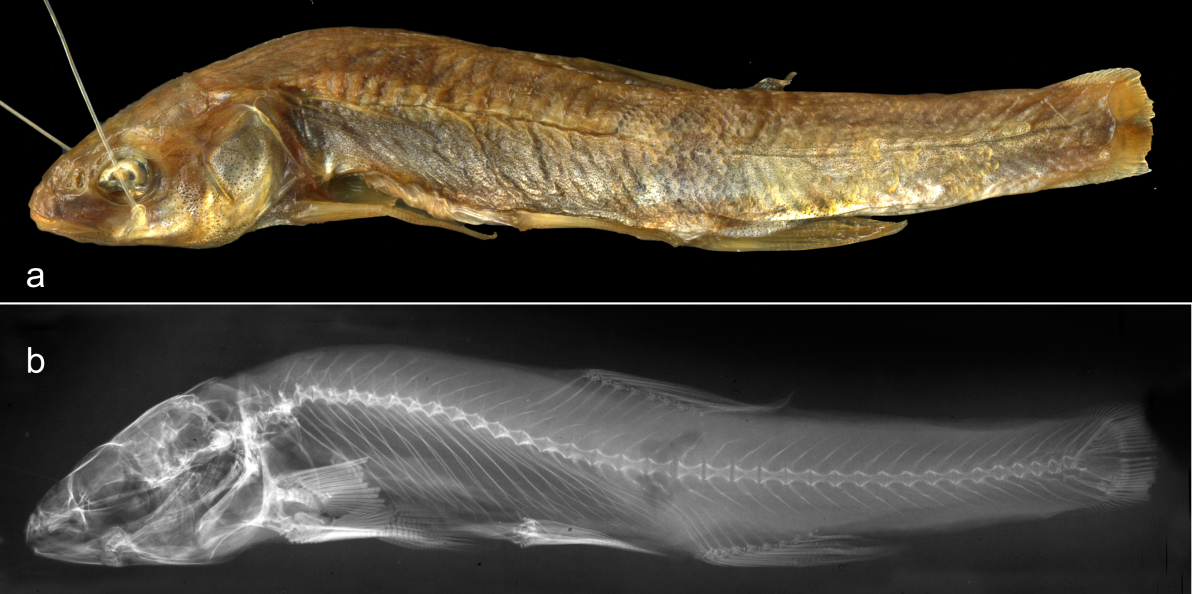


**Figure S5**: Photo (a) and x-ray (b) of the neotype *Phoxinus csikii* (NMW-51266/98673).

**
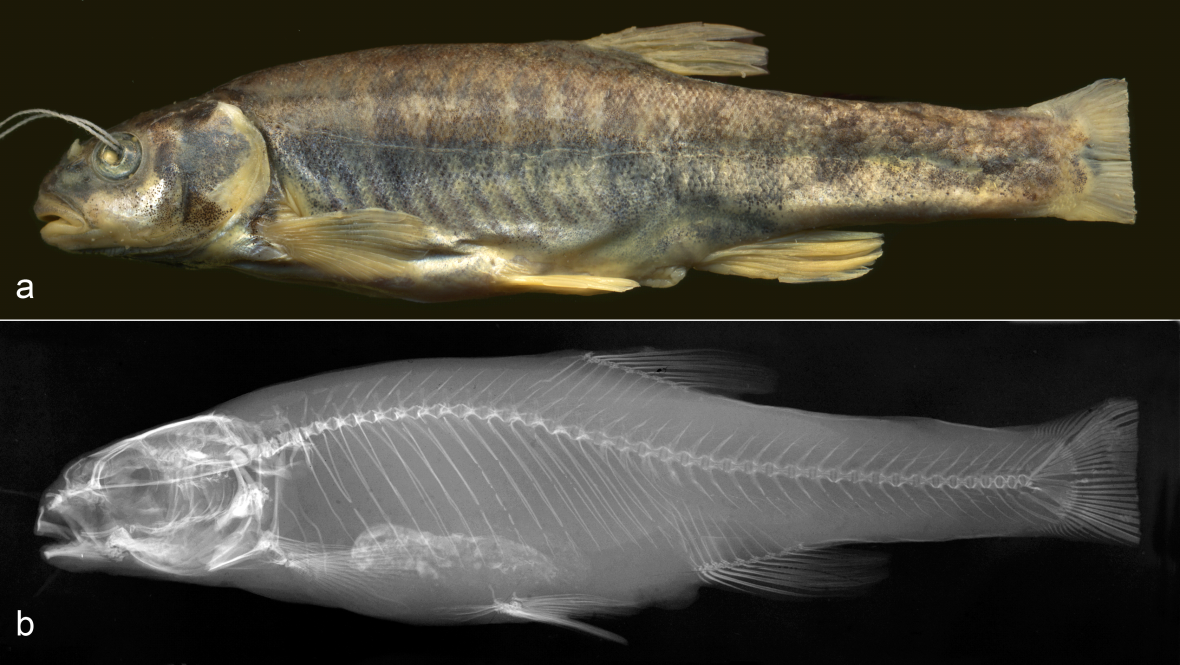
**

**References**

1. Zardoya R, Doadrio I: **Molecular evidence on the evolutionary and biogeographical patterns of European cyprinids**. *J Mol Evol* 1999, **49**(2):227-237.

2. Ward RD, Zemlak TS, Innes BH, Last PR, Hebert PD: **DNA barcoding Australia's fish species**. *Philosophical Transactions of the Royal Society B: Biological Sciences* 2005, **360**(1462):1847-1857.

3. Tamura K, Peterson D, Peterson N, Stecher G, Nei M, Kumar S: **MEGA5: molecular evolutionary genetics analysis using maximum likelihood, evolutionary distance, and maximum parsimony methods**. *Mol Biol Evol* 2011, **28**(10):2731-2739.

4. Palandačić A, Bravničar J, Zupančič P, Šanda R, Snoj A: **Molecular data suggest a multispecies complex of *Phoxinus* (Cyprinidae) in the Western Balkan Peninsula**. *Mol Phylogen Evol* 2015, **92**:118-123.

5. Chen W-J, Miya M, Saitoh K, Mayden RL: **Phylogenetic utility of two existing and four novel nuclear gene loci in reconstructing Tree of Life of ray-finned fishes: The order Cypriniformes (Ostariophysi) as a case study**. *Gene* 2008, **423**(2):125-134.

6. Quenouille B, Bermingham E, Planes S: **Molecular systematics of the damselfishes (Teleostei: Pomacentridae): Bayesian phylogenetic analyses of mitochondrial and nuclear DNA sequences**. *Mol Phylogen Evol* 2004, **31**(1):66-88.

7. Posada D: **jModelTest: phylogenetic model averaging**. *Mol Biol Evol* 2008, **25**(7):1253-1256.

8. Drummond AJ, Suchard MA, Xie D, Rambaut A: **Bayesian phylogenetics with BEAUti and the BEAST 1.7**. *Mol Biol Evol* 2012, **29**(8):1969-1973.

9. Baele G, Lemey P, Bedford T, Rambaut A, Suchard MA, Alekseyenko AV: **Improving the accuracy of demographic and molecular clock model comparison while accommodating phylogenetic uncertainty**. *Mol Biol Evol* 2012, **29**(9):2157-2167.

10. Baele G, Li WLS, Drummond AJ, Suchard MA, Lemey P: **Accurate model selection of relaxed molecular clocks in Bayesian phylogenetics**. *Mol Biol Evol* 2013, **30**(2):239-243.

11. Rambaut A, Suchard MA, Xie D, Drummond A: **Tracer v1.6**. In*.*; 2014.

12. Guindon S, Dufayard J-F, Lefort V, Anisimova M, Hordijk W, Gascuel O: **New algorithms and methods to estimate Maximum-Likelihood Phylogenies: assessing the performance of PhyML 3.0**. *Syst Biol* 2010, **59**(3):307-321.

13. Bazinet AL, Zwickl DJ, Cummings MP: **A gateway for phylogenetic analysis powered by grid computing featuring GARLI 2.0**. *Syst Biol* 2014, **63**(5):812-818.

14. Zwickl DJ: **Genetic algorithm approaches for the phylogenetic analysis of large biological sequence datasets under the maximum likelihood criterion**. University of Texas at Austin; 2006.

15. Sukumaran J, Holder M: **SumTrees: Phylogenetic tree Summarization. 4.0.0** In*.*: Dendrophy; 2015.

16. Sukumaran J, Holder MT: **DendroPy: a Python library for phylogenetic computing**. *Bioinformatics* 2010, **26**(12):1569-1571.

17. Puillandre N, Lambert A, Brouillet S, Achaz G: **ABGD, Automatic Barcode Gap Discovery for primary species delimitation**. *Mol Ecol* 2012, **21**(8):1864-1877.

18. Pons J, Barraclough TG, Gomez-Zurita J, Cardoso A, Duran DP, Hazell S, Kamoun S, Sumlin WD, Vogler AP: **Sequence-based species delimitation for the DNA taxonomy of undescribed insects**. *Syst Biol* 2006, **55**(4):595-609.

19. Zhang J, Kapli P, Pavlidis P, Stamatakis A: **A general species delimitation method with applications to phylogenetic placements**. *Bioinformatics* 2013, **29**(22):2869-2876.

20. Monaghan MT, Wild R, Elliot M, Fujisawa T, Balke M, Inward DJG, Lees DC, Ranaivosolo R, Eggleton P, Barraclough TG *et al*: **Accelerated species inventory on madagascar using coalescent-based models of species delineation**. *Syst Biol* 2009.

21. Ramler D, Palandačić A, Delmastro GB, Wanzenböck J, Ahnelt H: **Morphological divergence of lake and stream *Phoxinus* of Northern Italy and the Danube basin based on geometric morphometric analysis.** *Ecology and Evolution [in press]* 2016.

22. Tamura K, Kumar S: **Evolutionary distance estimation under heterogeneous substitution pattern among lineages**. *Mol Biol Evol* 2002, **19**(10):1727-1736.

23. Stephens M, Scheet P: **Accounting for decay of linkage disequilibrium in haplotype inference and missing-data imputation**. *The American Journal of Human Genetics* 2005, **76**(3):449-462.

24. Stephens M, Smith NJ, Donnelly P: **A new statistical method for haplotype reconstruction from population data**. *The American Journal of Human Genetics* 2001, **68**(4):978-989.

25. Librado P, Rozas J: **DnaSP v5: a software for comprehensive analysis of DNA polymorphism data**. *Bioinformatics* 2009, **25**(11):1451-1452.

26. Bandelt HJ, Forster P, Röhl A: **Median-joining networks for inferring intraspecific phylogenies**. *Mol Biol Evol* 1999, **16**(1):37-48.

**References for TableS1**

1. Eschmeyer WN, Fricke R, van der Laan R: **Catalog of Fishes: Genera, Species, References** [<http://researcharchivecalacademyorg/research/ichthyology/catalog/fishcatmainasp>] May 2017.

2. Kottelat M: **Three new species of *Phoxinus* from Greece and southern France (Teleostei: Cyprinidae)**. *Ichthyol Explor Freshwat* 2007, **18**(2):145-162.

3. Kottelat M, Freyhof J: **Handbook of European freshwater fishes**, vol. 13. Cornol, Switzerland: Publications Kottelat; 2007.

4. Bianco PG, De Bonis S: **A taxonomic study on the genus *Phoxinus* (Acthinopterigy, Cyprinidae) from Italy and western Balkans with description of four new species: *P. ketmaieri*, *P. karsticus*, *P. apollonicus* and *P. likai***. In: *Researches on Wildlife Conservation.* Edited by Bianco PG, de Filippo G, vol. 4. USA: IGF Publishing; 2015.

5. Heckel JJ: **Über einige neue, oder nicht gehörig unterschiedene Cyprininen, nebst einer systematischen Darstellung der Europäischen Gattungen dieser Gruppe**. *Annalen des Wiener Museums der Naturgeschichte* 1836, **1**:219-234.

6. Hankó B: **Halak [in Hungarian and German]**. *A Magyar Tudományos Akadémia Balkán-Kutatásainak tudományos eredményei* 1922, **1**:1-6.

7. Drensky, P. **[In Bulgarian, German summary]** Neue und seltene Fische aus Bulgarien. Trudove na Bulgarskogo Prirodoizpitatelno Druzhestvo Travaux de la Société Bulgare des Sciences Naturelles 1926, v. 12: 121-150.

8. Berg, L. S. **[In Russian, French subtitle]** Rapport sur une mission zoologique au Cascase en 1909. Zoologicheskago Muzeya Imperatorskoi Akademii Nauk 1910, v. 15: 153-170.

**References for TableS2**

1. Behrens-Chapuis S, Herder F, Esmaeili HR, Freyhof J, Hamidan NA, Özuluğ M, Šanda R, Geiger MF: **Adding nuclear rhodopsin data where mitochondrial COI indicates discrepancies – can this marker help to explain conflicts in cyprinids?** *DNA Barcodes* 2015, **3**(1):187-199.

2. Bergsten J, Englund M, Ericsson P: **A DNA key to all Swedish vertebrates** [<http://www.ncbi.nlm.nih.gov>] January 2016

3. Briolay J, Galtier N, Brito RM, Bouvet Y: **Molecular phylogeny of Cyprinidae inferred from cytochrome b DNA Sequences**. *Mol Phylogen Evol* 1998, **9**(1):100-108.

4. Geiger MF, Herder F, Monaghan MT, Almada V, Barbieri R, Bariche M, Berrebi P, Bohlen J, Casal-Lopez M, Delmastro GB *et al*: **Spatial heterogeneity in the Mediterranean Biodiversity Hotspot affects barcoding accuracy of its freshwater fishes**. *Molecular Ecology Resources* 2014, **14**(6):1210-1221.

5. Halacka K, Vetesnik L, Sanda R: **iBOL** [<https://www.ncbi.nlm.nih.gov/>] January 2016

6. Imoto JM, Saitoh K, Sasaki T, Yonezawa T, Adachi J, Kartavtsev YP, Miya M, Nishida M, Hanzawa N: **Phylogeny and biogeography of highly diverged freshwater fish species (Leuciscinae, Cyprinidae, Teleostei) inferred from mitochondrial genome analysis**. *Gene* 2013, **514**(2):112-124.

7. Knebelsberger T, Dunz AR, Neumann D, Geiger MF: **Molecular diversity of Germany's freshwater fishes and lampreys assessed by DNA barcoding**. *Molecular Ecology Resources* 2015, **15**(3):562-572.

8. Perea S, Böhme M, Zupančič P, Freyhof J, Šanda R, Özuluğ M, Abdoli A, Doadrio I: **Phylogenetic relationships and biogeographical patterns in Circum-Mediterranean subfamily Leuciscinae (Teleostei, Cyprinidae) inferred from both mitochondrial and nuclear data**. *BMC Evol Biol* 2010, **10**(1):265.

9. Strange RM, Mayden RL: **Phylogenetic relationships and a revised taxonomy for North American cyprinids currently assigned to *Phoxinus* (Actinopterygii: Cyprinidae)**. *Copeia* 2009, **2009**(3):494-501.

10. Thalinger B, Oehm J, Mayr H, Obwexer A, Zeisler C, Traugott M: **Molecular prey identification in Central European piscivores**. *Molecular Ecology Resources* 2015.
